# Derivation and transcriptional reprogramming of border-forming wound repair astrocytes after spinal cord injury or stroke in mice

**DOI:** 10.1038/s41593-024-01684-6

**Published:** 2024-06-21

**Authors:** Timothy M. O’Shea, Yan Ao, Shinong Wang, Yilong Ren, Amy L. Cheng, Riki Kawaguchi, Zechuan Shi, Vivek Swarup, Michael V. Sofroniew

**Affiliations:** 1 Department of Neurobiology, David Geffen School of Medicine, University of California, Los Angeles, CA USA; 2 Department of Biomedical Engineering, Boston University, Boston, MA USA; 3 Departments of Psychiatry and Neurology, University of California Los Angeles, Los Angeles, CA USA; 4 Department of Neurobiology and Behavior, University of California, Irvine, CA USA; 5 Institute for Memory Impairments and Neurological Disorders (MIND), University of California, Irvine, CA USA; 6 Present Address: Department of Orthopedics, Shanghai General Hospital, Shanghai Jiao Tong University, School of Medicine, Shanghai, PR China

**Keywords:** Astrocyte, Diseases of the nervous system

## Abstract

Central nervous system (CNS) lesions become surrounded by neuroprotective borders of newly proliferated reactive astrocytes; however, fundamental features of these cells are poorly understood. Here we show that following spinal cord injury or stroke, 90% and 10% of border-forming astrocytes derive, respectively, from proliferating local astrocytes and oligodendrocyte progenitor cells in adult mice of both sexes. Temporal transcriptome analysis, single-nucleus RNA sequencing and immunohistochemistry show that after focal CNS injury, local mature astrocytes dedifferentiate, proliferate and become transcriptionally reprogrammed to permanently altered new states, with persisting downregulation of molecules associated with astrocyte–neuron interactions and upregulation of molecules associated with wound healing, microbial defense and interactions with stromal and immune cells. These wound repair astrocytes share morphologic and transcriptional features with perimeningeal limitans astrocytes and are the predominant source of neuroprotective borders that re-establish CNS integrity around lesions by separating neural parenchyma from stromal and immune cells as occurs throughout the healthy CNS.

## Abstract (web summary)

The features of astrocytes surrounding CNS lesions are unclear. Here the authors show that after spinal cord injury or stroke in mice, mature astrocytes dedifferentiate, proliferate and are reprogrammed to adopt features of wound repair cells and form borders, re-establishing CNS integrity.

## Main

All organs share the ability to rapidly repair tissue lesions by generating newly proliferated cells that derive from stromal-cell, immune-cell and parenchymal-cell lineages. This multicellular proliferative wound response is a protective adaptation that limits tissue damage, sustains organ integrity and function, and is essential for organism survival^1^. In the CNS, astrocytes are key components of a multicellular proliferative wound response that is stimulated by tissue damage across a broad cross-section of CNS disorders including traumatic injury, stroke, infection, autoimmune inflammation and certain neurodegenerative diseases^2–6^. Understanding the derivation and temporally regulated proliferation, maturation, functions and potential failures of newly proliferated astrocytes during this wound response is fundamental to understanding and ameliorating the pathophysiology of many different CNS disorders.

Astrocytes are CNS parenchymal cells of neural progenitor cell origin^7^. They contiguously tile the entire CNS and provide multiple activities essential for CNS function in health and disease^2,8–12^. Astrocytes rarely divide in healthy adult CNS and exist in a state of potentially reversible cell cycle arrest (G0)^13,14^. Astrocytes respond to all forms of CNS injury and disease with molecular, structural and functional changes commonly referred to as astrocyte reactivity^2,6,15^. Notably, astrocyte reactivity can be either nonproliferative or proliferative^2,13,16^ and is tailored to different disorder contexts by multifactorial signaling mechanisms^17^.

Proliferative astrocyte reactivity occurs in response to overt CNS tissue damage and results in the formation of borders that surround tissue damaged by trauma, ischemia, infection, autoimmune inflammation, fibrosis, neoplasm, foreign bodies or pronounced neurodegeneration^4,13,16,18–25^. Multiple genetically targeted loss-of-function studies demonstrate that newly proliferated astrocyte borders serve essential functions that protect adjacent viable neural tissue, such that transgenic ablation or attenuation of border-forming astrocytes leads to impaired neural parenchymal wound repair with greater spread of destructive inflammation, larger fibrotic lesions, increased loss of neural tissue and impairment of neurological recovery^4,13,17,18,23,24,26–29^. Aging-associated perturbation of astrocyte proliferation and border formation is associated with increased loss of neurons and decreased functional recovery after spinal cord injury (SCI) in mice^30^. Despite the increasingly recognized importance of newly proliferated border-forming astrocytes, fundamental features of these cells are poorly understood. Here, we used a combination of transgenically targeted lineage tracing^31,32^, astrocyte-specific transcriptome analysis^17,33^, single-nucleus RNA sequencing (snRNA-seq) and immunohistochemical protein detection in mice to identify and selectively profile the transcriptional changes over time after traumatic injury of the predominant cellular source of newly proliferated astrocytes.

## Results

### Derivation of lesion border astrocytes

**Fig. 1:**
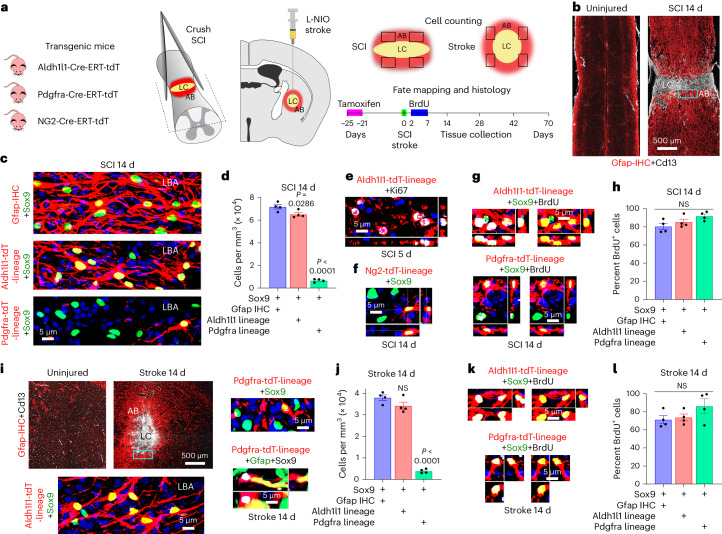
Lineage of border-forming astrocytes that surround CNS lesions. **a**, Lineage tracing procedures. **b**, Spinal cord, uninjured and after SCI, stained by immunohistochemistry for astrocytes (Gfap) or stromal cells (Cd13). **c**,**d**, Images (**c**) and cell counts (**d**) of Sox9-positive lesion border astrocytes (LBAs) plus Gfap-IHC or of lineage tracing with Aldh1l1-tdT or Pdgfra-tdT after SCI. **e**, Proliferating astrocytes labeled with Ki67. **f**, Staining for Sox9 plus Ng2-tdT. **g**,**h**, Newly proliferated BrdU-labeled astrocytes positive for Aldh1l1-tdT or Pdgfra-tdT after SCI (individual fluorescence channels are shown in Extended Data Fig. 1h). **i**,**j**, Striatum uninjured and after L-NIO stroke, with images (**i**) and cell counts (**j**) of Sox9-positive LBAs plus Gfap-IHC or of lineage tracing with Aldh1l1-tdT or Pdgfra-tdT after stroke. **k**,**l**, Newly proliferated BrdU-labeled astrocytes positive for Aldh1l1-tdT or Pdgfra-tdT after stroke. *n* = 4 mice per group. All graphs were evaluated with one-way ANOVA with Tukey’s post-hoc comparison. Bar graphs are mean values; error bars, s.e.m. *P* values are indicated on graphs. NS, nonsignificant; LC, lesion core; AB, astrocyte border. Source data

**Extended Data Fig. 1:**
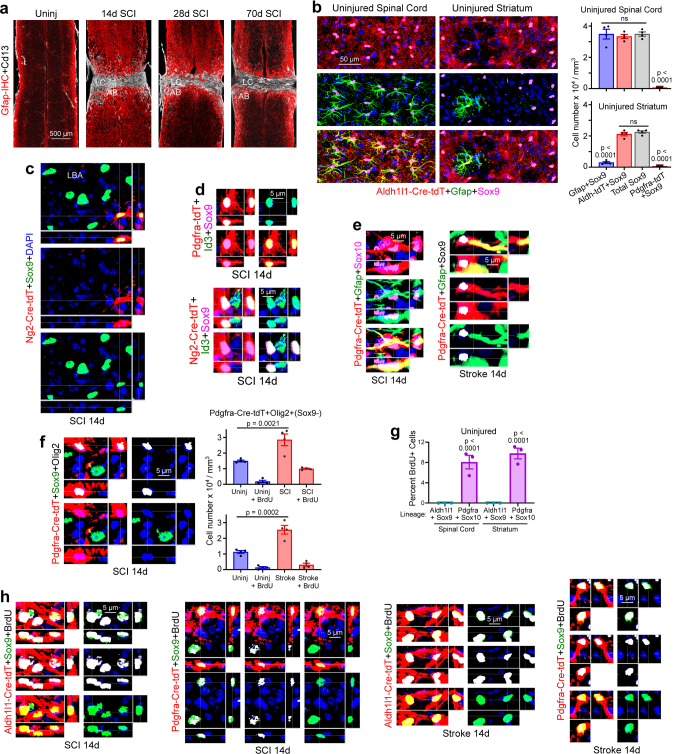
CreERT-tdT lineage tracing of border-forming astrocytes that surround CNS lesions. **a**. Survey images of horizontal sections of spinal cord uninjured and at progressive times after SCI stained by immunohistochemistry (IHC) for astrocytes (Gfap) or stromal cells (CD13), showing maturation and persistence of the astrocyte border (AB) over time. **b**. IHC and cell counts show that in uninjured spinal cord essentially all Aldh1l1-Cre-tdT labelled astrocytes also express Gfap and Sox9, whereas in uninjured striatum only about 13% do. In healthy spinal cord or striatum, no Gfap+ and Sox9+ astrocytes also detectably express Pdgfra-CreERT-tdT. n = 4 per group. **c**. NG2-Cre-tdT-positive OPC-lineage-derived Sox9-positive lesion border astrocyte (LBA) plus many Sox9-positive but NG2-Cre-tdT-negative Sox9-positive LBA after SCI. **d**. Examples of Pdgfra-Cre-tdT-positive NG2-Cre-tdT-positive OPC-lineage-derived LBA that express the transcription factor Id3. **e**. Examples of Pdgfra-Cre-tdT-positive OPC-lineage-derived LBA that express Gfap, Sox10, and Sox9. **f**. IHC and cell counts show that Pdgfra-Cre-tdT-positive OPCs that express Olig2 but not Sox9 are also present in the lesion border zone and that a portion of these OPCs proliferate and are labeled with BrdU after SCI or stroke. n = 4 per group. **g**. Cell counts show that in healthy cord or striatum there are no astrocytes detectably labelled with BrdU and dividing during a 6-day delivery period whereas, about 8 to 10% of OPC were BrdU-labelled and newly proliferated during the same delivery period. n = 3 per group. **h**. Examples of Aldh1l1-Cre-tdT-positive or Pdgfra-Cre-tdT-positive newly proliferated LBA that express Sox9 and are labelled with BrdU after SCI or stroke. All graphs evaluated with one way ANOVA with Tukey’s post hoc comparison. ns nonsignificant. All bar graphs are mean values +/- SEM. Source data

Studies from multiple laboratories implicate two main potential cellular sources for newly proliferated astrocytes around CNS injuries: local astrocytes^18–20,26,34^ and local oligodendrocyte progenitor cells (OPCs)^34–37^. Here, we used lineage tracing based on tamoxifen-regulated Cre-reporter expression^31^ to determine the proportional contributions of these cell types to newly proliferated border-forming astrocytes around hemorrhagic lesions after crush SCI or around ischemic lesions after forebrain stroke caused by infusion of N5-(1-iminoethyl)-l-ornithine (L-NIO, Fig. 1 and Extended Data Fig. 1). We targeted the reporter, tdTomato (tdT), to mature astrocytes by using Aldh1l1-CreERT^38^, and to OPCs by using either Pdgfra-CreERT-tdT^39^ or NG2-CreERT-tdT^40,41^, and induced temporary Cre expression with a 5-day regimen of tamoxifen dosing in healthy young adult (>8 week old) mice (Fig. 1a).

Newly proliferated astrocytes around CNS lesions organize into permanent, high cell density borders with overlapping cell processes that surround non-neural lesion cores of stromal and fibrotic tissue after SCI or stroke (Fig. 1b,c,i and Extended Data Fig. 1a)^13^. We quantified tdT-labeled, lineage-traced, border-forming astrocytes within representative 250 µm zones immediately adjacent to lesion core stromal tissue at 14 days after SCI or forebrain stroke (Fig. 1a–d,i,j), a timepoint by which border formation is largely complete^13^. As benchmarks against which to compare tdT labeling, we used Gfap and Sox9, which together label essentially all newly proliferated border-forming astrocytes around lesions^13,26,32^ (Fig. 1c,d,i,j).

In healthy adult spinal cord or striatum, essentially all astrocytes expressed Sox9 and Aldh1l1-CreERT-tdT, confirming previous reports^38^, and no astrocytes were detectably derived from OPCs as indicated by Pdgfra lineage tracing (Extended Data Fig. 1b). In uninjured spinal cord, all Sox9-positive and Aldh1l1-tdT-positive astrocytes expressed detectable Gfap, whereas in uninjured striatum, only about 13% did so (Extended Data Fig. 1b).

Lineage tracing showed that after both SCI and stroke, over 90% of Gfap plus Sox9-positive lesion border astrocytes also expressed Aldh1l1-CreERT-tdT, indicating that these cells derived from local mature astrocytes (Fig. 1c,d,i,j). Approximately 10% of Gfap plus Sox9-positive lesion border astrocytes expressed Pdgfra-CreERT-tdT and transcription factors Sox10 and Id3, indicating that these cells derived from local OPCs and exhibited molecular features of reactive astrocytes^17,42^ (Fig. 1c,d,f,i,j and Extended Data Fig. 1c–e). The proportion of OPC-derived border-forming astrocytes was essentially equivalent using NG2-CreERT-tdT lineage tracing (Fig. 1f and Extended Data Fig. 1c,d). In both SCI and stroke, about 75% of the Pdgfra-CreERT-tdT-positive cells in the lesion border zone were Olig2-positive but Gfap-negative and Sox9-negative OPCs (Extended Data Fig. 1f).

To identify newly proliferated cells, bromodeoxyuridine (BrdU) was administered during a 6-day period from 2 to 7 days after injury (Fig. 1a). In healthy adult spinal cord or striatum, no astrocytes were detectably BrdU-labeled, whereas about 8–10% of OPCs were (Extended Data Fig. 1g). At 5 days after injuries, lineage-traced astrocytes expressed the active proliferation marker Ki67 (Fig. 1e). Quantification of BrdU showed that at 14 days after either SCI or stroke, at least 75–85% of lesion border astrocytes that were derived from either astrocytes or OPCs were newly proliferated (Fig. 1g,h,k,l and Extended Data Fig. 1h), and this is probably a conservative estimate because BrdU was administered only once daily.

### Injury-induced transcriptional reprogramming

**Fig. 2:**
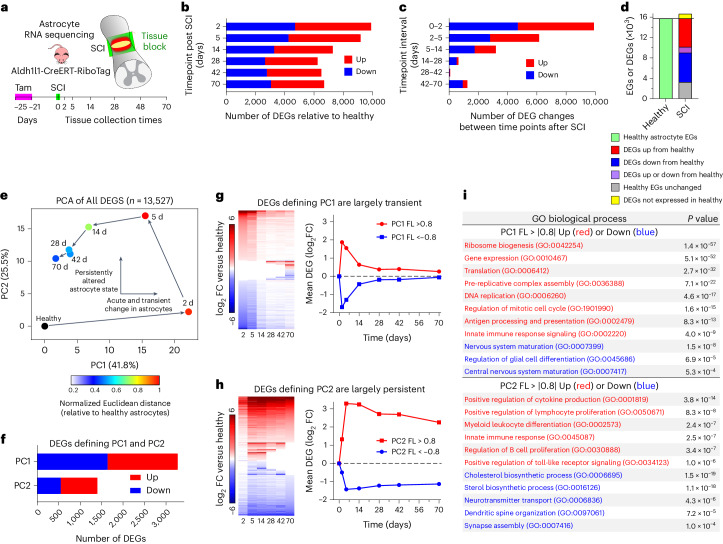
Temporal analysis of SCI-induced astrocyte transcriptional changes. **a**, Experimental design. **b**, Numbers of astrocyte DEGs significantly different (up or down; false discovery rate, FDR < 0.01) from uninjured healthy mice at different times. **c**, Number of astrocyte DEGs significantly (FDR < 0.01) changed between different timepoints. **d**, Number of healthy astrocyte expressed genes (EGs) compared with DEGs after SCI that are up, down or not regulated from healthy, or not expressed in healthy. **e**, PCA of all DEGs. **f**, Number of DEGs defining PC1 or PC2. **g**,**h**, Heatmaps and mean DEG log_2_ (fold change) (log_2_ FC) of DEGs defining PC1 (**g**) and PC2 (**h**). Heatmaps are arranged with the most upregulated DEGs at the top to most downregulated at the bottom on day 2 after SCI (**g**) and on day 70 (**h**). White spaces indicate no significant change. **i**, GO-BPs significantly upregulated (red) or downregulated (blue) as identified by unbiased evaluations of DEGs defined by PC1 or PC2. FL, PC factor loading. *P* values in **i** were calculated by two-sided Fisher’s exact test. *n* = 4 mice for uninjured and all post-SCI timepoints except day 2 (*n* = 5). Source data

**Extended Data Fig. 2:**
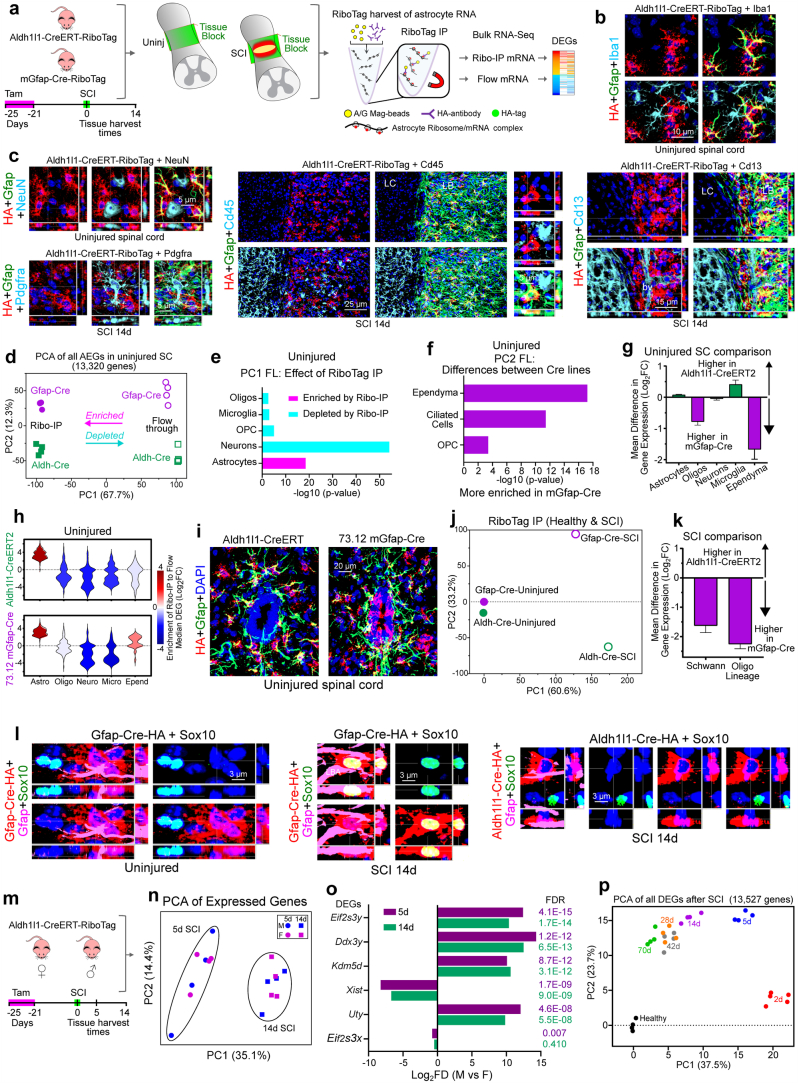
Comparison of transcriptional profiles obtained with tamoxifen-activated Aldh1l1-Cre-ERT-RiboTag or constitutive mGfap-Cre-RiboTag, and comparison of astrocyte transcriptional responses to SCI in female or male mice. **a**. Experimental design for bulk RNAseq of astrocyte expressed genes (EGs) obtained from uninjured mice by using transgenically targeted RiboTag using two different Cre lines, Ald1h1-CreERT or mGfap-Cre. **b,c**. Immunohistochemistry (IHC) of hemagglutinin (HA) derived from Aldh1l1-CreERT-RiboTag mice in uninured spinal cord and after SCI showed robust co-expression of HA and the astrocyte marker, Gfap, and no detectable overlap of HA staining with markers of microglia (Iba1), neurons (NeuN), OPCs (Pdgfra), stromal cells (Cd13) or white blood cells (Cd45) in either neural tissue or fibrotic lesion core tissue. **d**. Principal component analysis (PCA) of all EGs obtained by RNAseq of RiboTag immunoprecipitation (IP) of uninjured astrocyte EGs or flow through of non-astrocyte EGs from all other cells using the two different Cre lines. Note that both Cre lines show similar enrichment levels of astrocyte EGs by RiboTag-IP as demonstrated by PC1 which shows differences in astrocyte EGs relative to flow through EGs. Small differences between the two Cre lines are reflected in PC2. **e**. Cell type analysis of EGs whose factor loading (FC) determined PC1 demonstrates similar significant and substantial de-enrichment of genes associated with neurons, oligodendrocyte lineage cells and microglia using both Cre lines. Thus, in healthy spinal cord tissue, RNAseq of Aldh1l1-CreERT-RiboTag and mGfap-Cre-RiboTag gave similar transcriptional profiles that were highly significantly enriched for known astrocyte RNA transcripts and de-enriched for known transcripts associated with neurons, oligodendrocyte lineages or microglia. **f**. Cell type analysis of EGs whose FC determined PC2 indicates that differences between the two Cre lines was due to less de-enrichment of genes associated with ependyma, ciliated cells and OPC when using mGfap-Cre-RiboTag. **g,h**. Direct analysis of gene panels known to be associated with specific cell types showed pronounced and equal enrichment of astrocyte EGs and pronounced and essentially equal de-enrichment of neuronal and microglial EGs in both Cre lines, with greater de-enrichment of oligodendrocyte lineage genes using Aldh1l1-CreERT-RiboTag. Notably, ependymal genes were somewhat enriched in mGfap-Cre-RiboTag IPs. These differences could be explained by the expression of Gfap by multipotent radial glial progenitors that give rise to ependyma during development^86^. **i**. As expected, IHC demonstrated Gfap-Cre-RiboTag derived HA staining in many ependyma cells, which derive from Gfap-positive radial glia during development and express Cre for this reason. In contrast, HA was not detectable in ependyma in Aldh1l1-CreERT-RiboTag mice. **j**. PCA of RiboTag-IP RNAseq of uninjured astrocyte EGs or astrocytes after SCI comparing the two different Cre lines. Note that SCI induced similar levels of changes in astrocyte transcriptional profiles measured using the two Cre lines, as as demonstrated by PC1 of astrocyte DEGs. Differences between the two Cre lines are reflected in PC2. **k**. Cell type analysis of DEGs whose factor loading (FC) determined PC2 demonstrates significantly higher expression after SCI of DEGs associated with Schwann cells and oligodendrocyte lineage cells when using the mGfap-Cre line. This could be explained by the constitutive expression of mGfap-Cre-RiboTag and the acquisition after SCI of Gfap expression either by astrocytes derived from OPCs or by OPC-derived Schwann cells^34,87^. **l**. IHC for RiboTag (HA) shows that in uninjured mice, astrocytes that expressed mGfap-Cre-HA and Gfap protein are not co-labeled with Sox10, whereas after SCI, some mGfap-Cre-HA and Gfap-protein expressing cells astrocytes also express Sox10. **m**. Experimental design comparing SCI-induced transcriptional changes in female and male mice using the Aldh1l1-CreERT-RiboTag approach. **n**. PCA with unbiased clustering shows that transcriptional profiles cluster according to time after SCI with no detectable sex differences. **o**. Only two X-chromosome (Eif2s3x, Xist) and four Y-chromosome (Eif2s3y, Ddx3y, Kdm5d, Uty) genes exhibited sex differences in expression by astrocytes after SCI. **p**. PCA of FPKM values for all astrocyte DEGs obtained by RNAseq of Aldh1l1-CreERT-RiboTag IP of astrocytes harvested at various time points after SCI compared with astrocytes in uninjured spinal cords. Dots show values of n = 4 or 5 individual animals evaluated at each time point. All bar graphs are mean values +/- SEM. P-Values in (e&f) calculated by two-sided Fisher’s exact test. FDR values in (o) determined by quasi-likelihood F test with multiple testing correction using the Benjamini-Hochberg method. Source data

As mature Aldh1l1-expressing astrocytes are the predominant source of border-forming cells, we characterized their temporally dependent transcriptional changes after SCI. We used young adult (3–4 months old) male and female Aldh1l1-CreERT-RiboTag mice for hemagglutinin-positive ribosome immunoprecipitation and cell-specific transcriptome profiling of astrocytes^33,38^ in healthy, uninjured spinal cord and at 2, 5, 14, 28, 42 and 70 days after SCI (Fig. 2a, Extended Data Fig. 2a and Supplementary Data 1), spanning periods of proliferation, border formation and chronic border persistence^13,17,25^. Specificity of Aldh1l1-CreERT-RiboTag for astrocyte transcriptome analyses has been demonstrated in brain^38^ and was confirmed here for spinal cord (Extended Data Fig. 2a–k). Sequencing of mRNA from the flow-through solution after RiboTag immunoprecipitation was used to characterize gene expression by other local cells and enabled assessment of astrocyte enrichment (Extended Data Fig. 2a,d–k). Aldh1l1-CreERT-RiboTag gave equivalent astrocyte-enriched transcriptional profiles as mGfap-Cre-RiboTag^17,27^ but showed depleted genetic signatures originating from ependyma or OPC-derived cells that acquire Gfap expression after SCI; and astrocytes derived from OPCs after SCI had immunohistochemically detectable hemagglutinin expression in mGfap-Cre-RiboTag but not in Aldh1l1-CreERT-RiboTag spinal cords (Extended Data Fig. 2a,d–l). Consistent with previous reports after stroke^43^, we detected negligible sex-dependent transcriptomic differences in astrocytes after SCI, with four Y-chromosome and two X-chromosome genes detected as the only differentially expressed genes (DEGs) (false discovery rate of <0.01) across two independent post-SCI timepoints comparing eight female and eight male mice (Extended Data Fig. 2m–o).

Using Aldh1l1-CreERT-RiboTag (Astro-RiboTag) across all six post-SCI timepoints, a total of 13,527 unique DEGs were identified compared to healthy baseline levels, with more genes upregulated than downregulated at all timepoints (Fig. 2b and Supplementary Data 2). The most DEGs, 9,924, were detected at 2 days, with over 6,000 DEGs persisting at 28, 42 and 70 days (Fig. 2b). The greatest changes occurred between 0–2 and 2–5 days, with far fewer changes after 14 days (Fig. 2c).

Over 15,722 genes were identified as expressed by healthy spinal cord astrocytes. Of these genes, 37% (5,755) were primarily downregulated after SCI, 36% (5,598) were primarily upregulated, 7% (1,153) were dynamically regulated down or up at different timepoints and 20% (3,216) were not significantly different at any timepoint examined (Fig. 2d). Notably, 92% (12,506 out of 13,527) of the total DEGs identified across all times after SCI were expressed by astrocytes in healthy tissue (Fig. 2d). Induction of newly expressed genes that were not detectable in healthy astrocytes accounted for only 12% (889 out of 7640) of DEGs upregulated by astrocytes after SCI (Fig. 2d and Supplementary Data 2).

Principal component analysis (PCA) of the 13,527 DEGs identified across all timepoints revealed a clear temporal progression of astrocyte transcriptional responses, with two principal components that accounted for over two-thirds of the total system variation (Fig. 2e and Extended Data Fig. 2p). PC1, defining the most dominant effect, revealed acute and transient changes. DEGs with a PC1 factor loading of >|0.8| in the positive or negative direction peaked at 2–5 days after SCI before returning essentially to baseline healthy astrocyte levels by 28 days, with roughly equal numbers transiently upregulated or downregulated (Fig. 2f,g). DEGs defining PC2 increased quickly and largely persisted with mean values of >2 or less than −1 across the entire time course, with more upregulated (854) than downregulated (547) (Fig. 2f,h).

Unsupervised analysis of Gene Ontology Biological Processes (GO-BPs) showed that transiently upregulated DEGs defining PC1 were associated with the regulation of gene expression and translational, cell proliferation, innate immune signaling and antigen presentation, whereas transiently downregulated DEGs defining PC1 were associated with mature CNS structure and glia cell differentiation (Fig. 2i). Persistently upregulated DEGs defining PC2 were associated with cytokine production, innate and adaptive immune regulation and extracellular matrix (ECM) organization, whereas persistently downregulated DEGs defining PC2 were associated with cholesterol and lipid metabolism, neurotransmitter transport and synapse organization (Fig. 2i).

These findings demonstrate that local healthy mature Aldh1l1-expressing astrocytes undergo pronounced, temporally dependent transcriptional changes during border formation after SCI, including both transient and persisting changes. Remarkably, 88% of DEGs upregulated by astrocytes after SCI were already expressed at detectable levels by healthy astrocytes. Astrocyte border formation involves permanent transcriptional reprogramming, given that almost 50% of total DEGs persist at 70 days post SCI, including many genes not detectably expressed by healthy astrocytes. GO analyses parsed astrocyte transcriptional changes into profiles related to astrocyte dedifferentiation and proliferation, astrocyte reactivity, regulation of inflammation and immune signaling, wound healing and persisting border formation, examined in more detail below.

### Dedifferentiation, proliferation and loss of functions

**Fig. 3:**
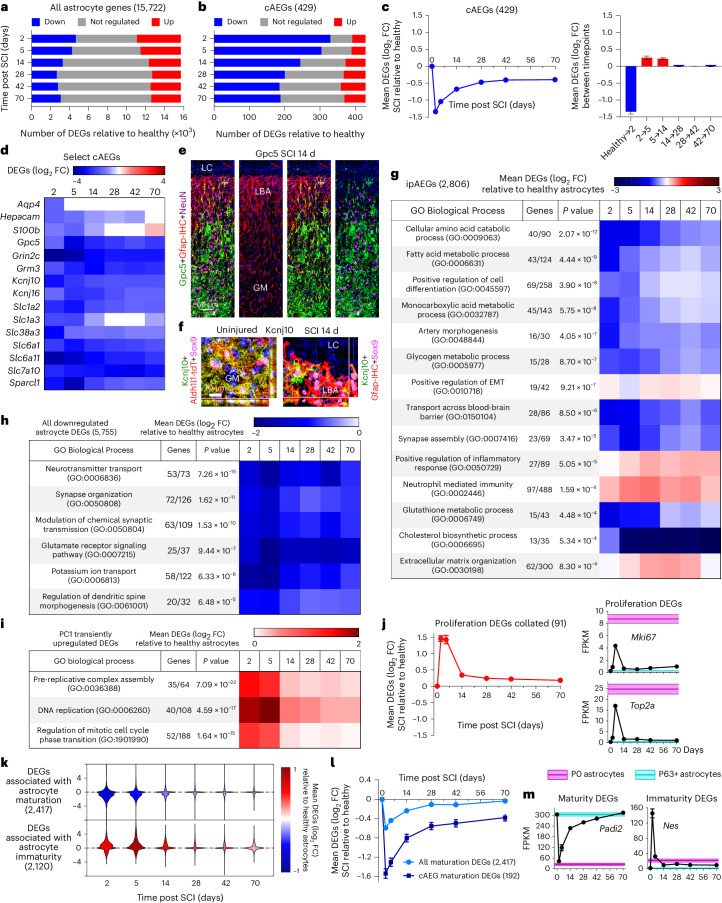
SCI-induced astrocyte dedifferentiation and proliferation. **a**, Numbers of up, down or nonsignificant changes (FDR < 0.01) in 15,722 genes expressed by uninjured astrocytes at different times after SCI. **b**, Numbers of up, down or nonsignificant changes (FDR < 0.01) in 429 cAEGs at different times after SCI. **c**, Mean log_2_ FC of downregulated cAEGs at different times after SCI relative to uninjured, and changes between individual timepoints. **d**, Heatmap of mean log_2_ FC of selected downregulated cAEGs at different times after SCI. **e**, Four different fluorescence channels illuminating different molecular markers within the same region to reveal the spatial distribution and colocalization of Gpc5 staining at LBA and GM astrocytes. Markedly Gpc5 immunoreactivities are reduced in LBAs compared with more distal gray matter (GM) astrocytes after SCI. **f**, Kcnj10 immunoreactivities in LBAs compared with more distal GM astrocytes after SCI. For individual fluorescence channels, see Extended Data Fig. 3c. **g**, Time course of mean expression changes after SCI of ipAEGs (see main text) associated with representative examples of the most significantly changed GO-BPs associated with ipAEGs. **h**, Highly downregulated GO-BPs associated with all downregulated astrocyte DEGs (whether enriched versus other cells or not). **i**, Time course after SCI of mean changes of DEGs associated with cell proliferation-related GO-BPs identified by PC1 in Fig. 2i. **j**, Time course after SCI of mean changes in all unsupervised 91 proliferation-related astrocyte DEGs examined in **i**, plus two additional canonical proliferation DEGs, *Mki67* and *Top2a*. **k**, Time courses after SCI of mean changes in consensus genes associated with astrocyte maturity or immaturity. **l**, Time courses after SCI comparing downregulation of all maturation-associated genes versus consensus genes expressed by mature astrocytes (cAEGs). **m**, Time courses after SCI of mean changes in two specific examples of DEGs, *Padi2* and *Nes*, associated with astrocyte maturity or immaturity, respectively. *n* = 4 mice for uninjured and all post-SCI timepoints except at day 2 (*n* = 5). Bar and line plots are mean values; error bars, s.e.m. *P* values in **g**–**i** calculated by two-sided Fisher’s exact test.

**Extended Data Fig. 3:**
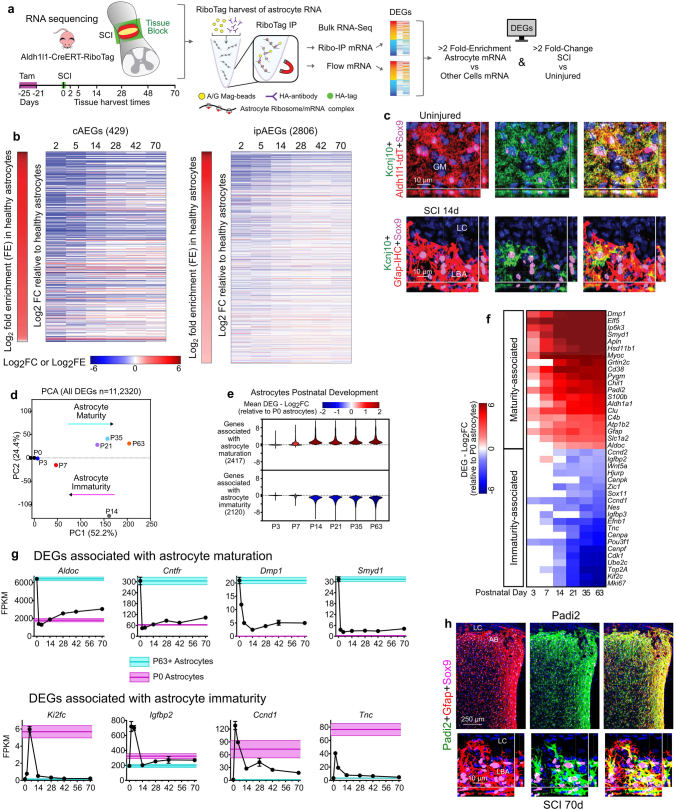
SCI-induced astrocyte dedifferentiation and proliferation, and expression profiles of genes associated with astrocyte immaturity or astrocyte maturity during development and after SCI. **a**. Experimental design for enrichment analysis of gene expression by astrocytes compared with other cells by comparing FPKM levels of astrocyte RNA precipitated via RiboTag with FPKM levels of RNA from other cells collected in non-precipitated flow through eluates. **b**. Heatmaps of log2 fold changes (Log_2_FC) in gene expression over time after SCI of 429 cAEGs and 2806 ipAEGs enriched in astrocytes by at least 2-fold and shown as log2 fold enrichment (Log_2_FE) compared with other cells. We confirmed that expression of all 429 cAEGs identified from the literature were enriched in our samples from health astrocytes by at least 2-fold and up to over 50-fold compared with non-astrocyte genes. **c**. Immunohistochemistry shows markedly reduced Kcnj10 (Kir4.1) protein immunoreactivity in lesion border astrocytes (LBA) compared with astrocytes in uninjured grey matter (GM). **d**. PCA of all DEGs showing DEG cohorts detected at various postnatal days (P) and thereby defining specific timepoints. **e**. Mean changes in genes associated with astrocyte maturity or immaturity at different postnatal (P) days. **f**. Heatmap of Log_2_FC changes in gene expression over time of selected genes associated with astrocyte maturity or immaturity at different postnatal (P0 or P63) days. **g**. Time course of expression changes (FPKM, fragments per kilobase of transcript per million reads) after SCI of selected genes associated with astrocyte maturity or immaturity compared with mean expression levels at different P0 or P63 days as markers of immature or mature levels, respectively. **h**. Immunohistochemistry of Padi2 protein at 70 days after SCI, an astrocyte maturity gene that is down regulated acutely after SCI and then returns to normal expression levels (see main Fig. 3m) and becomes prominently expressed in lesion border astrocytes (LBA). Line plots are mean values +/- SEM where n = 4 mice for uninjured and all post SCI timepoints except 2d which was n = 5.

We next defined more precisely how local mature astrocytes change in response to SCI. Of the 15,722 total genes expressed by healthy astrocytes (Fig. 2d), about 60% were upregulated or downregulated at 2 and 5 days, and about 35–40% from 28 through 70 days, with the rest remaining unchanged (Fig. 3a). To characterize astrocyte-enriched genes, we first examined a panel of 429 consensus healthy astrocyte-enriched genes (cAEGs) identified in at least five of eight published archival datasets^42^, which we confirmed as enriched in our healthy astrocytes by at least twofold and up to over 50-fold (Extended Data Fig. 3a,b and Supplementary Data 2). Nearly all cAEGs (97%; 417 out of 429) were DEGs after SCI and only 3% (12 out of 429) were not significantly different at any timepoint. Remarkably, 74% (317 out of 429) of cAEGs were primarily downregulated and only 11% (49 out of 429) were primarily upregulated, with 15% changing in either direction at different times (Fig. 3b). These changes were reflected in a pronounced decrease in mean expression of all 429 cAEGs, which had a negative peak at 2 days followed by a return towards baseline by 14 and 28 days, but with an overall downregulation persisting through 70 days (Fig. 3c). Notably, cAEGs with the highest enrichment in healthy astrocytes relative to other local cells were the most downregulated (Extended Data Fig. 3b).

Prominent cAEGs that were acutely downregulated and returned to baseline included the water channel (*Aqp4*), the calcium binding protein (*S100b*) and *Hepacam4*, which regulates astrocyte branching complexity^44^ (Fig. 3d). Prominent cAEGs that were persistently downregulated included transporters for glutamate (*Slc1a2* and *Slc1a3*), GABA (*Slc6a1 and Slc6a11*), glutamine (*Slc38ac*) and d-serine (*Slc7a10*), potassium channels (*Kcnj10* and *Kcnj16*), glutamate receptor subunits (*Grin2c* and *Grm*) and synapse modulating molecules (*Gpc5* and *Sparcl1*)^10,45^ (Fig. 3d). Immunohistochemistry (IHC) confirmed certain changes at the protein level and showed, for example, that many, but not all, lesion border astrocytes had low or undetectable levels of Gpc5 and Kcnj10 (Fig. 3e,f and Extended Data Fig. 3c).

To explore more broadly how local healthy astrocytes acutely changed functional states after SCI, we examined additional specific DEG cohorts. We first examined 2,806 genes identified by our RiboTag immunoprecipitation as significantly enriched in healthy astrocytes by at least twofold and up to 50-fold versus other local cells (Extended Data Fig. 3a,b and Supplementary Data 2). Similar to cAEGs, these immunoprecipitation astrocyte-enriched genes (ipAEGs) exhibited a preponderance of downregulation after SCI, particularly among those ipAEGs most highly enriched in healthy astrocytes (Extended Data Fig. 3b). We conducted unsupervised analysis of GO-BPs associated with significant changes among these 2,806 ipAEGs after SCI and tracked over time after SCI the mean expression of ipAEGs associated with representative examples of the most significantly changed GO-BPs. The majority of significantly altered GO-BPs were associated with downregulated ipAEGs related to cell differentiation, fatty acid metabolism, general metabolic processes, vascular morphogenesis, transport across the blood–brain barrier, synapse assembly, glutathione production and cholesterol production (Fig. 3g). The few GO-BPs associated with upregulated ipAEGs were related to epithelial-to-mesenchymal transition (EMT), immune functions and ECM reorganization.

We next examined GO-BPs associated with all 5,755 DEGs downregulated by astrocytes at any time after SCI, and again tracked over time the mean expression of DEGs associated with representative examples of the most significantly changed. This analysis further confirmed a pronounced acute and persistent attenuation in the expression by astrocytes of genes associated with astrocyte–neuron interactions, neurotransmitter transport, synapse organization, synaptic transmission and potassium regulation (Fig. 3h).

Cell dedifferentiation can be associated with proliferation^46^. Past^13^ and present Ki67 and BrdU evaluations (Fig. 1e,g,h,k,l) and GO-BP analysis (Fig. 2i) indicate pronounced astrocyte proliferation starting around 2 days after SCI. We tracked over time after SCI changes among genes associated with three of the most significantly upregulated cell proliferation-related GO-BPs identified by PCA (Figs. 2i and 3i,j). Astrocyte DEGs associated with each of these GO-BPs, and the mean expression of the unsupervised 91 proliferation-related genes associated with all three GO-BPs, were highly upregulated by local astrocytes at 2 and 5 days and returned to near baseline levels by 14 days (Fig. 3i,j and Supplementary Data 2).

Dedifferentiation and proliferation of local healthy Aldh1l1-expressing astrocytes after SCI suggested a potential return to an immature or progenitor-like state^47^. We compared astrocyte DEGs after SCI with DEG panels positively or negatively associated with astrocyte maturation during postnatal development derived by PCA of transcriptomes from healthy astrocytes at postnatal days (P) from P0 to P63 (Extended Data Fig. 3d–h). By 2 and 5 days after SCI, astrocytes had markedly downregulated mean expression of 2,417 genes associated with maturity and upregulated mean expression of 2,120 genes associated with immaturity, and these changes returned to essentially baseline levels by 28 days, with a modest upregulation of some immaturity genes persisting to 70 days (Fig. 3k and Extended Data Fig. 3d–h). Of the 429 cAEGs, 192 were positively associated with the progression toward maturity, and their mean expression declined and remained persistently low after SCI (Fig. 3l,m). Notably, healthy P0 astrocytes expressed high levels of *Mki67* and *Top2a* associated with active proliferation (Fig. 3j), whereas mature astrocytes in healthy CNS are proliferation-dormant and exist in a potentially reversible G0 state (Extended Data Fig. 1g). They required two or more days after injury to express these genes and never reach fragments per kilobase of transcript per million mapped reads (FPKM) levels of healthy proliferating P0 astrocytes (Fig. 3j).

These findings demonstrate that local healthy mature Aldh1l1-expressing astrocytes respond acutely to SCI with downregulation of most astrocyte-enriched genes and a transient phase of proliferation and immaturity. This is followed by a return of many transcriptional features of mature astrocytes but with persisting differences, including a persistent downregulation of molecules associated with astrocyte–neuron interactions such as maintenance of extracellular neurotransmitter and ion homeostasis, and synapse organization and function. These findings point towards persistent transcriptional reprogramming of newly proliferated lesion border astrocytes to new and different functional states.

### Reactivity and gain of functions

**Fig. 4:**
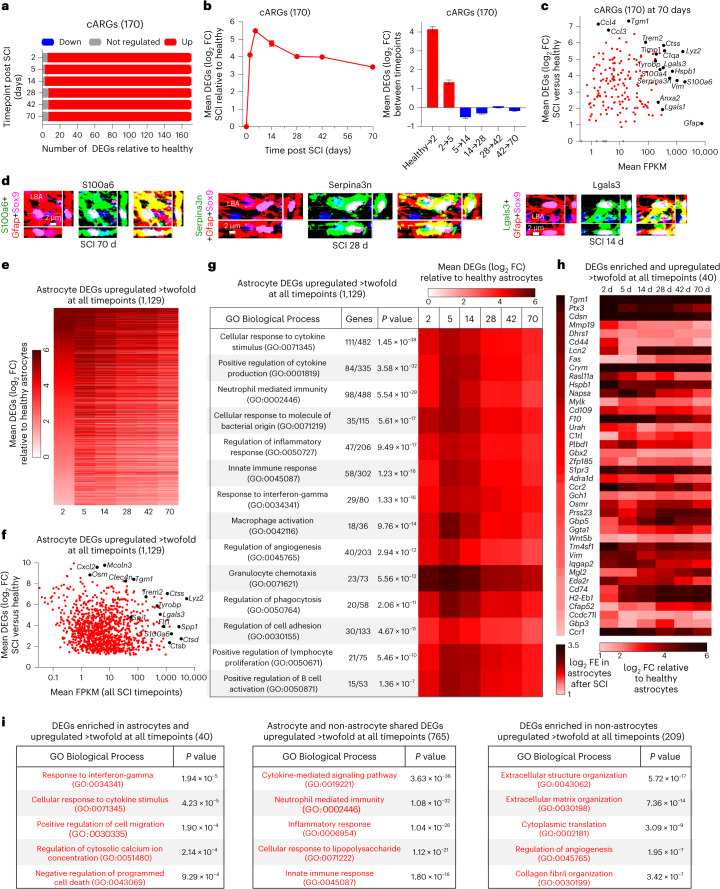
SCI-induced astrocyte transcriptional changes associated with reactivity and gains of functions. **a**, Numbers of up, down or nonsignificant (FDR < 0.01) changes in 170 cARGs at different times after SCI. **b**, Mean log_2_ FC of upregulated cARGs at different times after SCI and changes between individual timepoints. **c**, Scatterplot showing log_2_ FC and FPKM of cARGs at 70 days after SCI. Selected examples of highly expressed and highly upregulated DEGs are labeled. **d**, Examples of cARGs with high levels of protein immunoreactivity in LBAs. **e**, Heatmap of 1,129 astrocyte DEGs upregulated at least twofold at all times after SCI. **f**, Scatterplot showing log_2_ FC and FPKM of 1,129 astrocyte DEGs upregulated at least twofold at all times after SCI. **g**, Top GO-BTs associated with 1,129 astrocyte DEGs upregulated at least twofold at all times after SCI. **h**, Heatmaps of log_2_ (fold enrichment) (log_2_ FE) and log_2_ FC of 40 DEGs enriched in astrocytes by a mean of at least twofold compared with other cells and upregulated by at least twofold at all timepoints after SCI. **i**, Top GO-BPs associated either with 40 DEGs expressed more highly by astrocytes than other cells, or with 765 DEGs expressed at similar levels by astrocytes and other cells, or with 209 DEGs expressed more highly by other cells. *n* = 4 mice for uninjured and all post-SCI timepoints except at day 2 (*n* = 5). Bar and line plots are mean values; error bars, s.e.m. *P* values in **g** and **i** were calculated by two-sided Fisher’s exact test.

**Extended Data Fig. 4:**
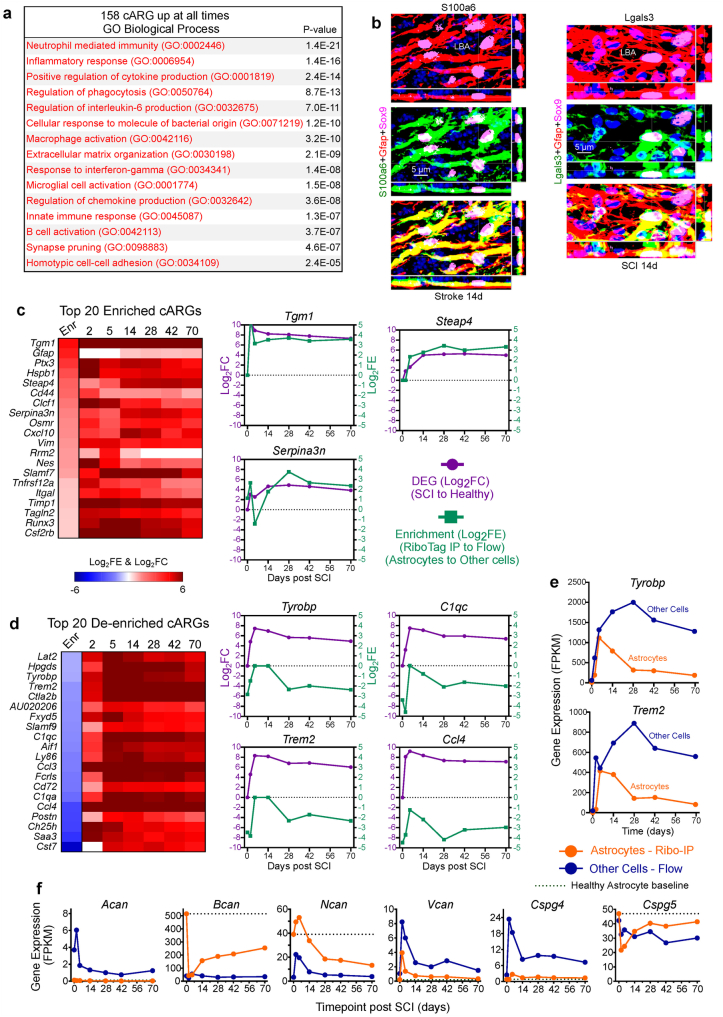
Reactivity DEGs. **a**. Top GO Biological Processes associated with 158 cARGs upregulated at all times after SCI. **b**. Immunohistochemistry of S100a6 and Lgals3 protein in lesion border astrocytes (LBA) after stroke (S100a6) or SCI (Lgals3). Note that while S100a6 is present in the processes of most LBA, Lgals3 is present in some but not others. **c,d**. Heatmaps of mean log_2_FC after SCI of 20 cARGs that are either most enriched (**c**) or most de-enriched (**d**) in astrocytes compared with other cells; graphs compare log_2_FC with mean log_2_FE in selected examples. **e**. Comparison of expression (FPKM) of *Tyrobp* and *Trem2* by astrocytes and other cells at different times after SCI. Note that between 5, 14 and 28 days after SCI, expression levels of *Tyrobp* and *Trem2* increase markedly in other cells while simultaneously decreasing markedly in astrocytes. Such a divergence in DEG levels would not be observed if *Tyrobp* and *Trem2* levels in astrocytes were an artifact caused by the non-specific contamination of RiboTag IP samples by highly expressed transcripts derived from other cells. **f**. Expression (FPKM) by astrocytes and other cells of chondroitin sulphate proteoglycans (CSPGs) after SCI. Note that the prototypical CSPG, *Acan*, is not detectably expressed by astrocytes at any time after SCI, and *Vcan* and *Csp4* were far more highly expressed by non-astrocytes. Only *Bcan* and *Ncan* are more highly expressed by astrocytes, but *Bcan* expression declines after SCI to levels below healthy, and although *Ncan* expression increases at 5 days after SCI, by 28 days it also declined to levels lower than in healthy astrocytes. Line plots are mean values +/- SEM where n = 4 mice for uninjured and all post SCI timepoints except 2d which was n = 5. P-Values in (a) calculated by two-sided Fisher’s exact test.

To look for potential new functions adopted by newly proliferated and reprogrammed astrocytes after SCI, we first examined changes in 170 consensus astrocyte reactivity genes (cARGs) derived from a meta-analysis of six archival datasets from multiple laboratories^42^ (Supplementary Data 2). All 170 cARGs were upregulated on at least one timepoint after SCI, 93% (158 out of 170) were upregulated at all timepoints and 92% (157 out of 170) were detectably expressed by healthy astrocytes (Fig. 4a). Mean cARG expression increased 16-fold by 2 days after SCI, increased to over 50-fold by 5 days and then declined moderately but remained persistently elevated by over eightfold at 70 days (Fig. 4b), consistent with reprogramming to an essentially permanent reactive state after SCI. Notably, 41 of the top 50 GO-BPs most significantly associated with cARGs upregulated at all timepoints involved regulation of inflammation, while other upregulated GO-BPs included phagocytosis, ECM organization, synapse pruning and homotypic cell–cell adhesion (Extended Data Fig. 4a). cARGs that were upregulated by 14 days after SCI and remained persistently and highly upregulated at 70 days included well-studied cARGs such as *Gfap*, *Vim* and *Lgals3*, as well as molecules that appear in multiple reactive astrocyte RNA evaluation studies such as *S100a6, Serpina3n, Lyz2, Lcn2, Hsbp1, C1qa, Tyrobp, Trem2, Tgm1*, *Ccl3* and *Ccl4* (Fig. 4c). IHC confirmed protein expression by border-forming astrocytes for many of these molecules after SCI and stroke, and revealed that whereas certain proteins such as Gfap and S100a6 were readily detectable in essentially all Sox9-positive lesion border astrocytes, many proteins, such as Lgals3 and others discussed below, were highly expressed in some lesion border astrocytes that were intermingled with other astrocytes with low or no detectable expression (Fig. 4d and Extended Data Fig. 4b).

Given that the injury response involves many cell types^3^, we compared the expression of the same genes by astrocytes and other cells (Extended Data Fig. 3a). Many highly upregulated cARGs (Fig. 4c) were also highly enriched in other cells, such as *Tgm1*, *Steap4*, *Serpina3n* and others (Extended Data Fig. 4c). Nevertheless, many highly upregulated cARGs were de-enriched in astrocytes relative to other cells, such as *Tyrobp*, *Trem2*, *C1qc, Ccl4* and others, indicating that although these genes were used by reactive astrocytes, they were also more prominently used by other cells (Extended Data Fig. 4d). Notably, astrocytes and other cells often exhibited different temporal patterns of upregulation or downregulation of these DEGs after SCI, including changes in opposite directions at different times, suggesting potentially different roles exerted by different cell types at different times and supporting the specificity of the expression of these transcripts by astrocytes (Extended Data Fig. 4e).

We next examined 1,129 DEGs upregulated by at least twofold at all timepoints after SCI (Fig. 4e,f and Supplementary Data 2). Most of these DEGs had peak expressions at 5 days and remained markedly elevated for at least 70 days (Fig. 4e). We then tracked over time the mean expression changes of astrocyte DEGs associated with representative examples of the top GO-BPs associated with these 1,129 DEGs (Fig. 4g). Remarkably, all top 15 and 40 of the top 50 GO-BPs involved regulation of inflammation, including both innate and adaptive immune responses such as cytokine production, granulocyte chemotaxis, macrophage activation and lymphocyte regulation (Fig. 4g). Other persistently upregulated GO-BPs included regulation of angiogenesis, phagocytosis and cell adhesion (Fig. 4g).

To identify functions that might be preferentially associated with transcriptional changes in reactive astrocytes, we compared GO-BPs associated with upregulated DEGs enriched either in astrocytes or in other cells. As noted above, ipAEGs exhibited a mean downregulation of DEGs but included some upregulated DEGs (Extended Data Fig. 3b), of which only 40 were also enriched in astrocytes by over twofold (Fig. 4h). The top GO-BPs associated with these 40 upregulated and astrocyte-enriched ipAEGs included response to interferon-gamma and cytokines, cytosolic calcium regulation and negative regulation of programmed cell death (Fig. 4i). The top GO-BPs associated with DEGs enriched and upregulated in other cells (non-astrocytes) were reorganization of ECM and angiogenesis, and the top GO-BPs associated with DEGs similarly upregulated by both astrocytes and non-astrocytes were cytokine signaling and regulation of innate immunity and inflammation (Fig. 4i). These findings indicated that gene expression changes in astrocytes and non-astrocytes contribute to overlapping as well as to differing biological functions after SCI. Notably, ECM reorganization was more prominently associated with DEGs deriving from non-astrocytes (Fig. 4i). Chondroitin sulfate proteoglycans (CSPGs) are ECM components that have received prominent attention in SCI and have previously been attributed primarily to astrocytes. Nevertheless, three of six CSPG transcripts were more highly expressed by non-astrocytes at all timepoints after SCI, and not a single CSPG transcript exhibited prominent or persistent upregulation by astrocytes above baseline healthy levels (Extended Data Fig. 4f).

These findings show that as newly proliferated astrocytes reprogram after SCI, they exhibit both transient and persistent upregulation of many different DEGs. Analyses of GO-BPs associated with persistently upregulated DEGs point towards reprogrammed astrocytes adopting new functions after SCI that contribute to innate immune responses, regulation of inflammation, control of infection, debris phagocytosis and regulation of angiogenesis, which we examined further.

### Immune regulation, antigen presentation, antimicrobial defense

**Fig. 5:**
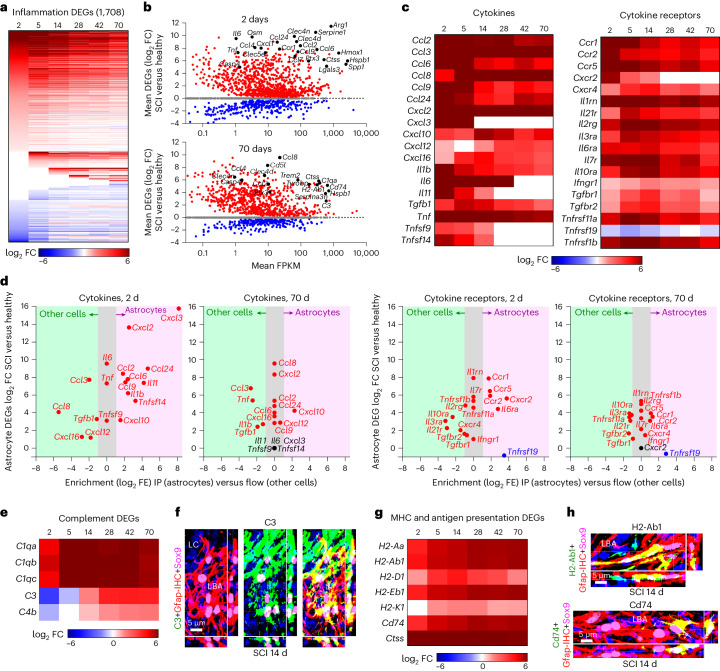
Innate and adaptive immune signaling, complement signaling and antigen presentation. **a**, Heatmap of 1,708 consensus inflammation-associated DEGs upregulated or downregulated by astrocytes at different days after SCI. **b**, Scatterplots showing log_2_ FC and FPKM of 1,708 astrocyte consensus inflammation-associated DEGs at 2 or 70 days after SCI. **c**, Heatmaps of mean log_2_ FC of selected cytokines and cytokine receptors expressed by astrocytes after SCI. **d**, Comparison of relative enrichment and expression levels of various cytokines and cytokine receptors by astrocytes and by other cells at 2 and 70 days after SCI. **e**, Heatmap of mean log_2_ FC of selected complement-related DEGs expressed by astrocytes after SCI. **f**, High levels of C3 protein immunoreactivity in LBAs after SCI. **g**, Heatmap of mean log_2_ FC of selected DEGs associated with antigen presentation and expressed by astrocytes after SCI. **h**, High levels of H2-Ab1 or Cd74 protein immunoreactivity in scattered individual LBAs intermingled among many negative LBAs after SCI. *n* = 4 mice for uninjured and all post-SCI timepoints except at day 2 (*n* = 5).

The most prominent gain-of-function GO-BPs associated with astrocyte transcriptional reprogramming after SCI were related to innate and adaptive immune responses such as neutrophil recruitment, microglial and macrophage activation, antigen processing and presentation, antimicrobial activity, and B cell and T cell recruitment (Figs. 2i, 3g and 4g). To examine the time course of individual DEG changes related to these functions, we compiled a list of 2,766 unique genes associated with these GO-BPs. Of these 2,766 genes, 1,708 exhibited changes by astrocytes after SCI consisting primarily of acute upregulation at 2–14 days, with some returning towards baseline levels and many exhibiting long-term upregulation for up to 70 days (Fig. 5a,b and Supplementary Data 3).

**Extended Data Fig. 5:**
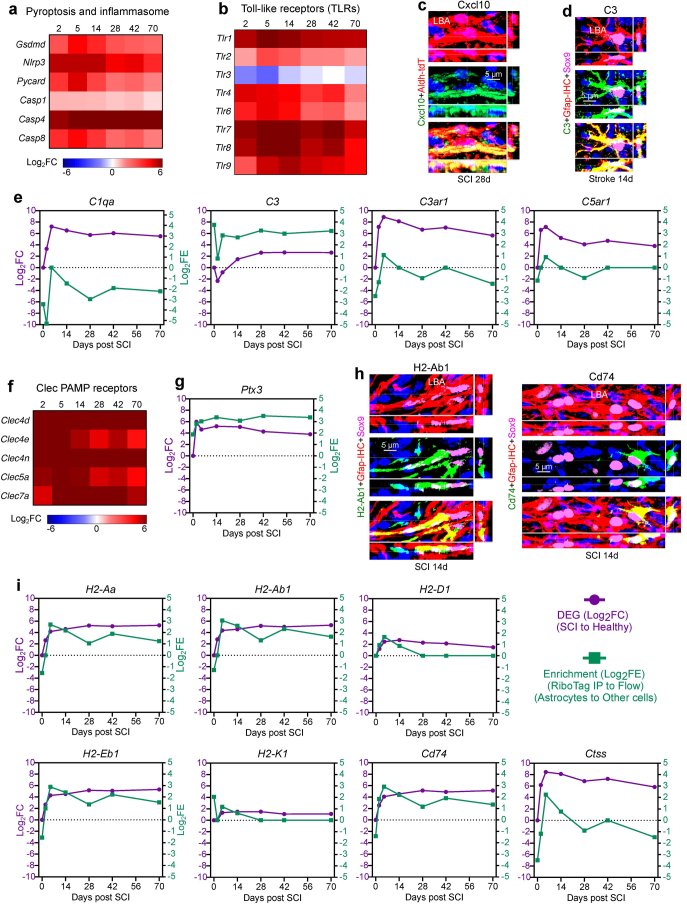
Innate and adaptive immune regulatory signaling, and contributions to antimicrobial defense and antigen presentation. **a,b**. Heatmaps of Log_2_FC of DEGs associated with astrocyte pyroptosis and inflammasome generation (**a**) or Toll-like receptors (**b**) at different days after SCI. **c,d**. Immunohistochemistry of Cxcl10 protein after SCI (**c**) and C3 protein after stroke in lesion border astrocytes (LBA). (**d**). **e**. Graphs compare changes in log_2_FC with changes in mean log_2_FE after SCI of selected complement related DEGs. **f**. Heatmaps of Log_2_FC of *Clec* DEGs encoding pathogen-associated molecular patterns (PAMPs) receptors. **g**. Graph showing both upregulation of expression (log_2_FC) and enrichment (log_2_FE) in border astrocytes of antimicrobial *Pitx3*. **h**. Immunohistochemistry showing intense expression of H2-Ab1 protein or C74 protein in scattered border astrocytes while other nearby astrocytes do not exhibit detectable levels. **i**. Graphs compare changes in log_2_FC with changes in mean log_2_FE after SCI of selected DEGs associated with antigen presentation.

In addition to multiple cytokines, chemokines and their receptors, notable immune regulatory DEGs acutely upregulated by astrocytes at 2 days after SCI included *Arg1*, *Ctss*, *Hmox1*, *Hspb1*, *Serpine1* and *Lgals3;* persistently upregulated immune regulatory DEGs at 70 days included *Cd74*, *H2-Ab1*, *H2-Eb1*, *H2-Aa*, *C1qa*, *C3*, *Trem2*, *Tyrobp*, *Serpina3n*, *Lcn2* and *Ctss* (Fig. 5b,c). Cytokines included many pro-inflammatory but also some anti-inflammatory (*IL11*, *Tgfb* and *IL6*) molecules (Fig. 5b,c). Most cytokines peaked rapidly followed by some decline and moderate long-term persistence (*Ccl2*, *Ccl3*, *Cxcl2*, *Tnf* and *Spp1*), some cytokines peaked acutely and were downregulated to baseline (*Cxcl3*, *IL6* and *Il11*) and a few gradually increased their expression over time (*Ccl8*) (Fig. 5b,c). Upregulated cytokine and chemokine receptors included *Ccr1*, *Ccr5*, *1l1rn* and *Il10ra* (Fig. 5c). We also found evidence consistent with astrocyte pyroptosis and inflammasome generation^48^ with upregulation of *Dsdmd*, *Nlrp3*, *Pycard*, *Casp1*, *Casp4* and *Casp8* (Extended Data Fig. 5a). In addition, in agreement with findings from multiple laboratories^16^, we found that multiple toll-like receptors were markedly upregulated by astrocytes from 2 to 14 days, including *Tlr1*, *Tlr4*, *Tlr6*, *Tlr7*, *Tlr8* and *Tlr9*, and some of these remained persistently elevated (Extended Data Fig. 5b), consistent with astrocyte involvement in diverse innate immune responses.

We compared expression of the same cytokines and receptors in astrocytes and other cells (Fig. 5d). At 2 days after SCI, *Cxcl3* and *Cxcl2* were the most highly upregulated and highly enriched in astrocytes, suggesting potentially unique roles for astrocytes compared with other cells (Fig. 5d). Additional cytokines that were both highly upregulated and enriched in astrocytes at 2 days included *Ccl2*, *Ccl6*, *Ccl9*, *Ccl24*, *Cxcl10*, *Il1b*, *Il11* and *Tnfsf14* (Fig. 5d). Notably, many cytokines were upregulated by astrocytes but were nevertheless more highly expressed by other cells at 2 days, such as *Ccl3*, *Ccl8* and *Cxcl16* (Fig. 5d). By 70 days, *Cxcl3* had returned to baseline in astrocytes, whereas *Cxcl2*, *Ccl8*, *and Ccl2* remained upregulated at levels comparable to other cells and only *Cxcl10* was persistently upregulated and enriched in astrocytes, as confirmed also by IHC (Fig. 5d and Extended Data Fig. 5c). At 2 days after SCI, upregulated cytokine receptors enriched in astrocytes included *Ccr1*, *Ccr2*, *Ccr5*, *Cxcr2* and *Il6ra*, whereas at 70 days, various receptors were upregulated but were present at levels comparable to other cells (Fig. 5d). These findings suggest the potential for certain unique, and many shared, cytokine-related functions among astrocytes and other cell types after SCI.

Astrocytes also upregulated genes associated with antimicrobial defense in complement pathways and antigen presentation. *C1qa*, *C1qb* and *C1qc* were upregulated by over 50-fold from 5 days through at least 70 days after SCI (Fig. 5e). Microbiocidal^49,50^ *C3* and *C4b* were initially downregulated at 2 and 5 days but were then persistently upregulated through 70 days (Fig. 5e), and immunoreactive C3 protein was prominently detected in lesion border astrocytes that interfaced with non-neural lesion core cells in SCI and stroke (Fig. 5f and Extended Data Fig. 5d). Notably, although C1q was upregulated by astrocytes, it was more highly expressed by other cells, whereas C3 was both upregulated and enriched in astrocytes (Extended Data Fig. 5e). Complement receptors *C3ar1* and *C5ar1* were also upregulated by over 50-fold and were initially enriched in astrocytes but became equally or more highly expressed by other cells by 28 days and longer after SCI (Extended Data Fig. 5e).

Additional antimicrobial defense DEGs included *Clec4d*, *Clec4n*, *Clec5a* and *Clec7a*, which encode pathogen-associated molecular pattern receptors for viruses, bacteria and fungi, as well as *Ptx3*, *Lyz2* and *Lgals3* (Figs. 4c,d and 5b and Extended Data Fig. 5f,g). Multiple DEGs associated with antigen presentation and major histocompatibility complex (MHC) class II were not only prominently upregulated from 5 through 70 days after SCI but in many cases were enriched in astrocytes compared with other cells, including *H2-Aa, H2-Ab1, H2-Eb1* and *Cd74* (Fig. 5g,h and Extended Data Fig. 5h,i). Notably, immunoreactive protein for these major histocompatibility complex class II and related molecules was high in scattered lesion border astrocytes but not detectable in others (Fig. 5h and Extended Data Fig. 5h), consistent with specialized expression among some but not other astrocytes.

These findings show that the transcriptional reprogramming of newly proliferated astrocytes after SCI includes both transient and persistent changes in many DEGs associated with multiple innate and adaptive immune functions, including diverse pro-inflammatory and some anti-inflammatory cytokine signaling, antigen presentation and antimicrobial defense. The persistent upregulation of these DEGs points towards a long-term contribution to immune preparedness by border-forming astrocytes around persisting CNS lesions.

### EMT, wound repair, cell adhesion and border maturation

**Fig. 6:**
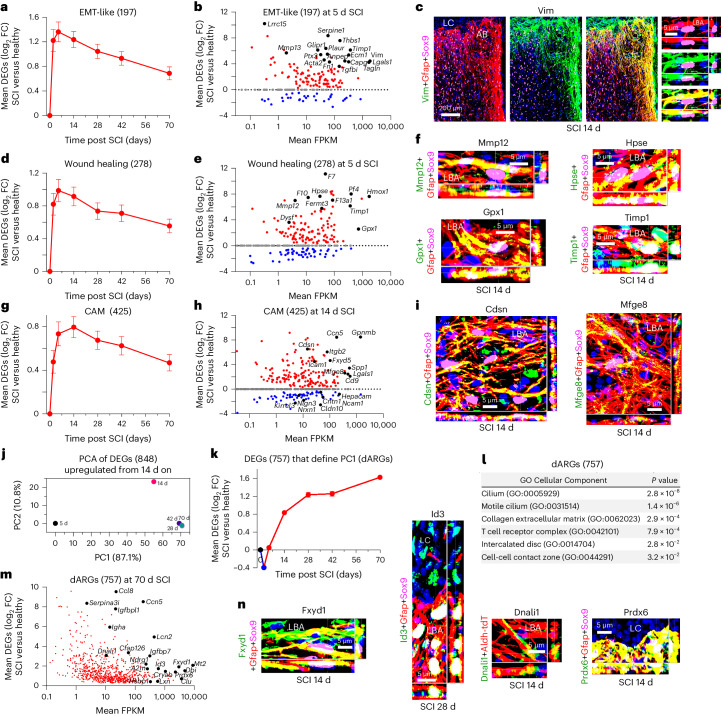
Wound healing, cell adhesion changes and border maturation. **a**, Mean log_2_ FC of a panel of 197 EMT-like DEGs at different times after SCI relative to uninjured. **b**, Scatterplot showing log_2_ FC and FPKM of EMT-like DEGs at 5 days after SCI. **c**, High protein immunoreactivity of the canonical EMT marker, Vim, in LBAs. **d**, Mean log_2_ FC of a panel of 278 wound-healing-associated DEGs at different times after SCI. **e**, Scatterplot showing log_2_ FC and FPKM of wound healing DEGs at 5 days after SCI. **f**, High protein immunoreactivity of selected wound-healing-associated molecules in LBAs. **g**, Mean log_2_ FC of a panel of 425 cell adhesion molecule (CAM) DEGs at different times after SCI. **h**, Scatterplot showing log_2_ FC and FPKM of CAM DEGs at 14 days after SCI. **i**, High protein immunoreactivity of selected CAMs in LBAs. **j**, PCA of 848 DEGs upregulated from 14 days onwards after SCI relative to 5 days after SCI. **k**, Mean log_2_ FC of the 757 dARGs that define PC1. **l**, Top GO Cellular Components associated with the 757 dARGs. **m**, Scatterplot showing log_2_ FC and FPKM of dARGs at 70 days after SCI. **n**, Examples of high protein immunoreactivity in LBAs of selected dARGs. *n* = 4 mice for uninjured and all post-SCI timepoints except at day 2 (*n* = 5). Bar and line plots are mean values; error bars, s.e.m. *P* values in **l** were calculated by two-sided Fisher’s exact test.

**Extended Data Fig. 6:**
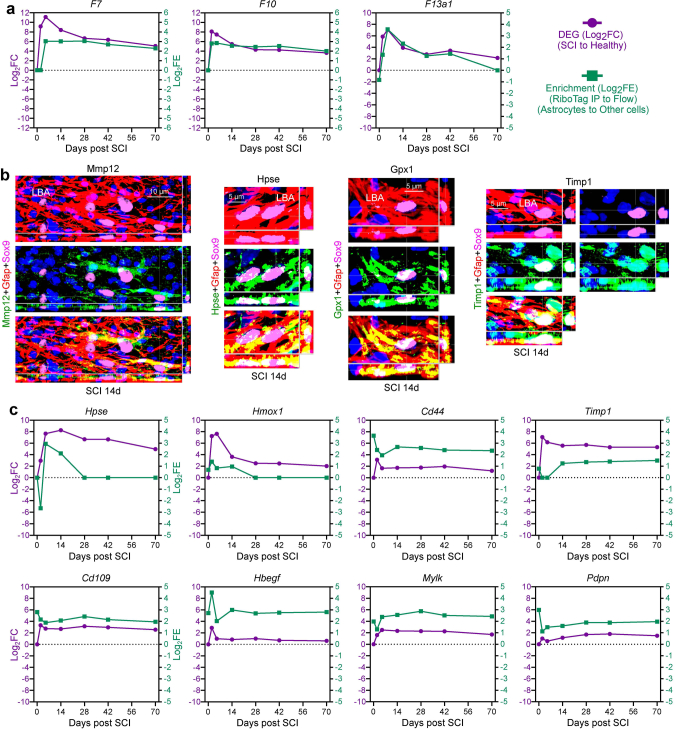
Wound healing associated DEGs. **a**. Graphs compare changes in log_2_FC with changes in mean log_2_FE after SCI of selected coagulation factor associated DEGs. **b**. Immunohistochemistry of Mmp12, Hpse, Gpx1 and Timp1 proteins in lesion border astrocytes (LBA) after SCI. Note in Mmp12 image that two astrocytes express high levels of immunoreactive Mmp12, whereas the majority of astrocytes in the field of view do not. **c**. Graphs compare changes in log_2_FC with changes in mean log_2_FE after SCI of selected diverse wound healing associated DEGs.

Another prominently upregulated GO-BP associated with ipAEGs was ‘regulation of EMT’ (Fig. 3g). We examined a panel of 197 consensus EMT-associated DEGs adapted from Msigdb gene sets^42^ and found a rapid acute increase in mean expression that peaked at 2 to 5 days and declined thereafter but remained persistently elevated above baseline, including prototypical EMT genes such as *Vim, Fn1* and *Acta2* (Fig. 6a–c and Supplementary Data 3). Previous studies have identified EMT genes in astrocyte responses to SCI^42,51,52^, and EMT-associated DEGs in adult cells have been implicated in wound healing^52–54^. Therefore, we conducted a hypothesis-driven analysis of 278 DEGs associated with the GO-BP ‘wound healing’ (GO:0042060) and also found a rapid acute increase in mean expression in astrocytes at 2 to 5 days after SCI that declined gradually but remained persistently elevated (Fig. 6d and Supplementary Data 3). Notable and unexpected wound-healing-associated DEGs that were highly expressed by astrocytes sub-acutely from 2–14 days after SCI included coagulation factors *F7*, *F10* and *F13a1*, which were upregulated over 50-fold to 100-fold and enriched in astrocytes compared with other cells (Fig. 6e and Extended Data Fig. 6a); heparin-degrading and hemostasis molecules *Pf4*, *Hpse* and *Fermt*; heme-degrading enzyme *Hmox1*; phagocytosis promoting *Cd44*; membrane repair *Dysf*; tissue remodeling *Mmp12* and *Timp1*; free-radical scavenger *Gpx1*; and others including *Hbegf*, *Cd109*, *Cd151, Mylk* and *Pdpn* (Fig. 6e,f and Extended Data Fig. 6b). Remarkably, many of these DEGs were both upregulated and enriched in astrocytes compared with other cells, suggesting unique and important wound repair functions for reactive astrocytes (Extended Data Fig. 6c), and immunoreactive protein for certain molecules such as Mmp12 was high in some lesion border astrocytes and low or not detectable in others (Extended Data Fig. 6b).

**Extended Data Fig. 7:**
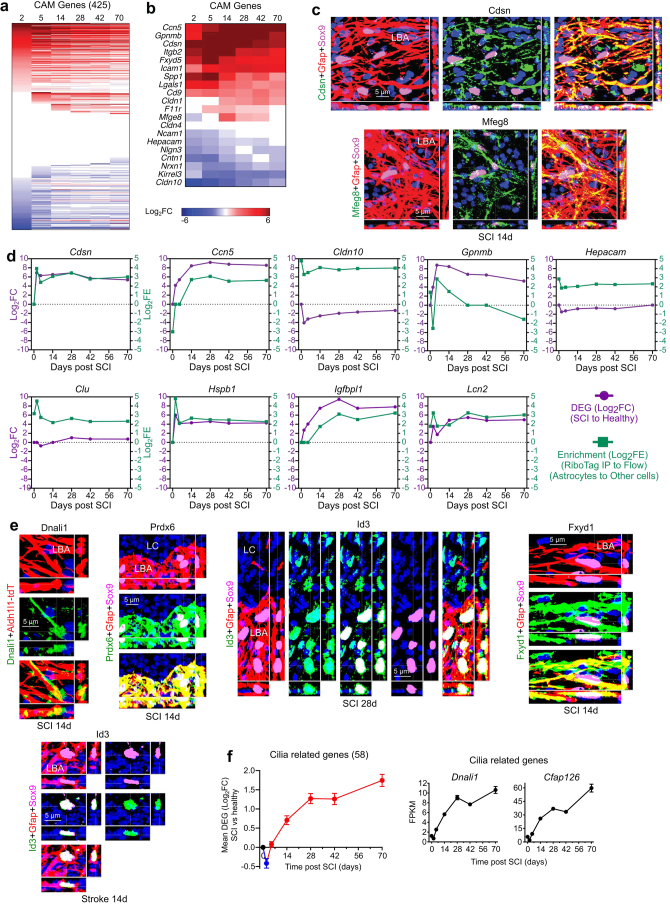
Cell adhesion molecules (CAMs) and delayed astrocyte reactivity DEGs (dARGs). **a,b**. Heatmaps of Log_2_FC of all (**a**) and selected (**b**) CAM-associated DEGs at different times after SCI. **c**. Immunohistochemistry of Cdsn and Mfeg6 CAM proteins in lesion border astrocytes (LBA) after SCI. **d**. Graphs comparing changes in log_2_FC with changes in mean log_2_FE after SCI of selected dARGs. **e**. Immunohistochemistry of Dnali1, Prdx6, Id3 and Fxyd1 proteins in LBA after SCI or stroke. **f**. Graphs show delayed mean upregulation after SCI of 58 cilia related DEGs and representative examples of *Dnali1* and *Cfap126*. Line plots in (f) are mean values +/- SEM where n = 4 mice for uninjured and all post SCI timepoints except 2d which was n = 5.

As noted above, newly proliferated lesion border astrocytes downregulate the domain-associated cell adhesion molecule (CAM) *Hepacam*^44^ (Fig. 3d), and as they mature do not adopt individual domains but instead reorganize with highly overlapping and intermingled cell processes (Fig. 1c). Therefore, we looked for changes in DEGs associated with cell–cell interactions by examining a panel of 425 CAMs and cell–cell interaction-associated genes compiled from gene set enrichment analysis, GO and the literature. After SCI, astrocytes exhibited an overall early and persisting mean upregulation in expression of CAM-associated DEGs, but with about 40% of individual DEGs exhibiting upregulation, 40% downregulation and 20% no change (Fig. 6g–i, Extended Data Fig. 7a and Supplementary Data 3). Notable upregulated CAM-associated DEGs included *Ccn5*, *Gpnmb* and *Spp1* involved in integrin binding; *Lgals1* and *Fxyd5* involved in matrix adhesion; *Cdsn*, *Cd9* and *Mfge8* involved in cell–cell adhesion; and *Itgb2* and *Icam1* involved in leukocyte adhesion (Fig. 6h,i and Extended Data Fig. 7b,c). Notable downregulated CAMs included *Hepacam*, *Cldn10*, *Cntn1*, *Kirrel3*, *Ncam1*, *Nrxn1* and *Nlgn3* involved in homophilic astrocyte–astrocyte interactions and interactions with neurons (Fig. 6h and Extended Data Fig. 7b). Many of these CAMs were enriched in astrocytes compared with other cells (Extended Data Fig. 7d).

The DEGs and associated GO-BPs examined thus far peaked acutely after SCI. To identify potential features of border-forming astrocytes that might emerge after the acute period, we next examined 848 DEGs that were upregulated from 14 to 70 days. PCA revealed a prominent difference defined by PC1 between 5 days and the more chronic timepoints, and a smaller difference defined by PC2 between 14 and 28–70 days (Fig. 6j). Factor analysis of the 757 DEGs defining PC1 revealed a cohort of delayed astrocyte reactivity genes (dARGs) whose mean expression declined acutely and then increased and remained high from 14 to 70 days (Fig. 6k and Supplementary Data 3). These dARGs exhibited GO Cellular Components associated with ciliated cells, ECM interactions and intercalated disk cell–cell contact interactions (Fig. 6l). Notable dARGs at 70 days after SCI included the transcription factor *Id3*; proteinase inhibitors *A2m, Lxn* and *Serpina3i*; the neuroprotective endozepine *Dbi*^55^; antioxidants *Mt1*, *Mt2* and *Prdx6*; ion transport regulator *Fxyd1*; chaperone *Clu*, multifunctional *Igfbpl1*, antimicrobial *Lcn2*, *Nrdg1* and *Igha*; heat shock protein *Cryab*; and cilia-associated proteins including *Dnali1* and *Cfap126* (Fig. 6m,n and Extended Data Fig. 7e,f). Many of these dARGs were enriched in astrocytes (Extended Data Fig. 7d). Protein expression for various dARGs was confirmed by IHC and Id3, Fxyd1 and Prdx6, in particular, were robustly detected in most if not all lesion border astrocytes (Fig. 6n and Extended Data Fig. 7e). Delayed expression of *Id3* and various cilia-related genes including *Dnali1* and *Cfap126* (Fig. 6m and Extended Data Fig. 7f) is consistent with their established roles in proliferation arrest and promotion of astrocyte differentiation of immature progenitor-like cells and points towards their possible involvement in ending the transient phase of proliferation and immaturity observed in the initial post-injury response^14,56–58^.

Together, these findings show that the transcriptional reprogramming of newly proliferated astrocytes after SCI equips astrocytes with the potential to contribute to important wound healing processes, including hemostasis, coagulation and breakdown of heme in hemorrhagic CNS wounds, free-radical scavenging, and tissue and matrix remodeling. Remarkably, many of the associated DEGs were not only upregulated but were also enriched in astrocytes compared with other cells, suggesting unique and important wound repair functions for newly proliferated reactive astrocytes. The findings also reveal major changes in CAM expressions (both up and down) by lesion border astrocytes around CNS lesions, indicating major readjustments in their homophilic and heterophilic cell–cell interactions consistent with their major change in morphology from cells with unique non-overlapping cellular domains^44^ to cells with highly overlapping and intertwined cellular processes (Fig. 1c).

### Phenotypic features of mature lesion border astrocytes

**Fig. 7:**
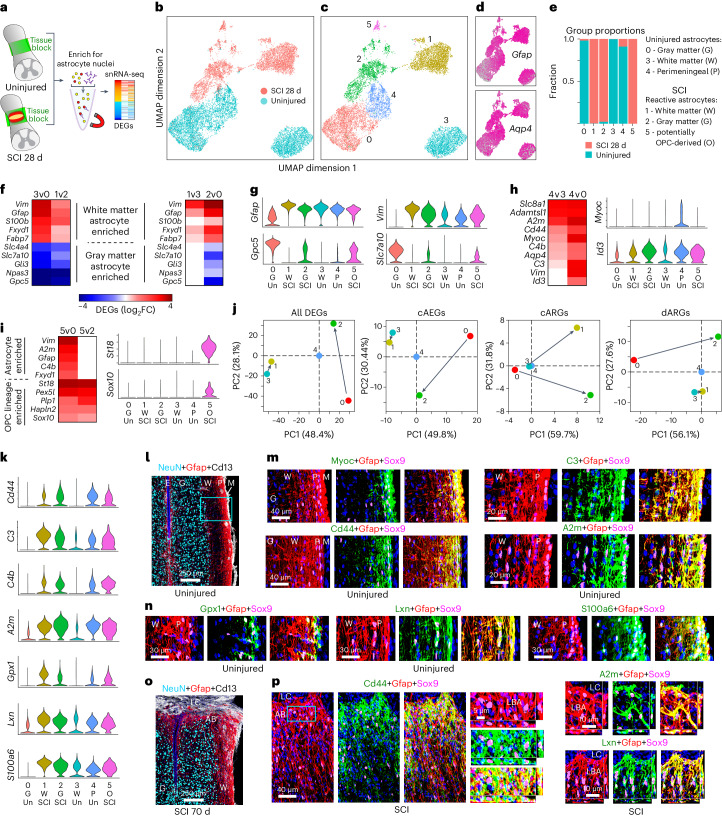
Shared and distinct molecular features of uninjured astrocytes and mature LBAs. **a**, snRNA-seq procedures. **b**,**c**, UMAP clusters of astrocyte nuclei. **d**, *Gfap* and *Aqp4* expression across clusters. **e**, Proportion of nuclei from uninjured or SCI mice per cluster. **f**,**g**, Heatmaps (**f**) and violin plots (**g**) comparing marker genes enriched in gray (G) or white matter (W) astrocytes across different clusters. P, perimeningeal; O, OPC-derived; Un, uninjured. **h**,**i**, Heatmaps and violin plots of genes enriched in perimeningeal astrocytes (**h**) or in OPC-derived astrocytes (**i**). **j**, PCA comparing changes in different gene cohorts across clusters after SCI. **k**, Violin plots of genes enriched in both perimeningeal and reactive astrocytes compared with uninjured. **l**–**n**, IHC of proteins enriched in uninjured perimeningeal astrocytes and LBAs after SCI. **l**,**o**, Survey images of uninjured (**l**) and SCI (**o**). **m**,**n**, Details from boxed region in **l** showing proteins enriched in perimeningeal astrocytes. **p**, Proteins enriched in LBAs. E, ependyma; M, meninges.

**Extended Data Fig. 8:**
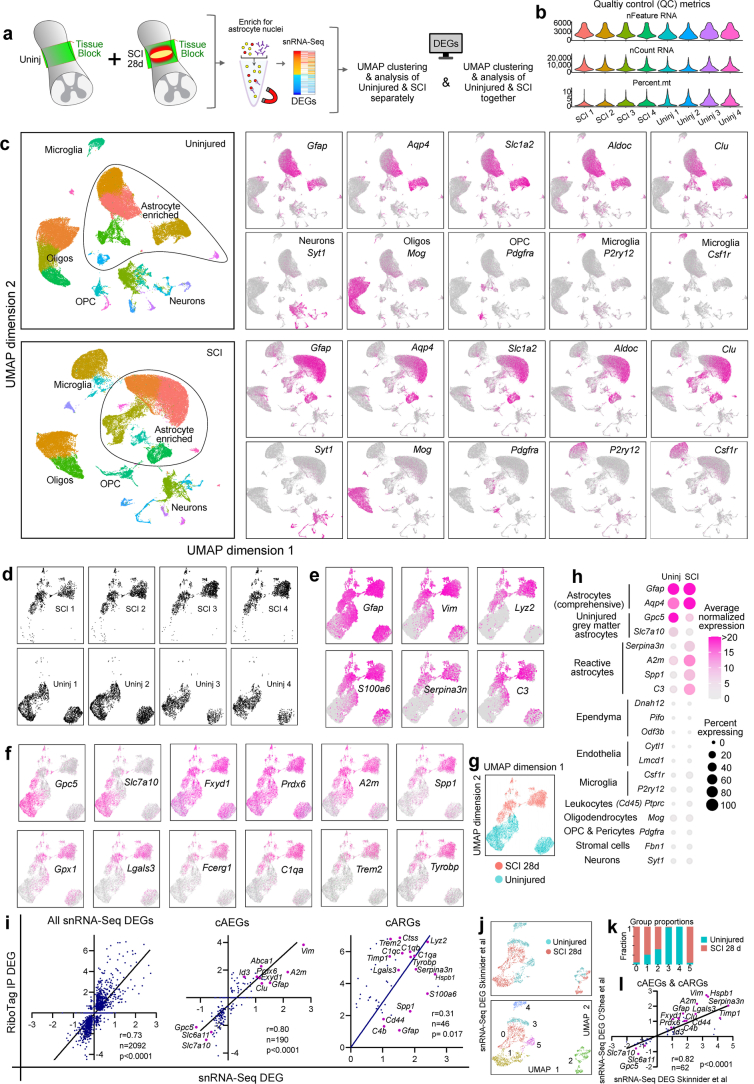
Single nucleus RNA sequencing (snRNAseq). **a**. Experimental design for snRNAseq of the same region of thoracic spinal cord tissue from uninjured mice (n = 4) and mice at 28 days after SCI (n = 4). **b**. Quality control (QC) features of 4 uninjured (Uninj) and 4 SCI replicates prior to clustering. **c**. UMAP clusters of all nuclei. Clusters were annotated as enriched for Astrocytes, Neurons, Oligodendrocytes (Oligos), Oligodendrocyte Precursor Cells (OPC), or Microglia based on panels of multiple cell-type specific marker genes. Custers identified as astrocyte-enriched were extracted for re-clustering and further extraction based on cohorts of multiple astrocyte specific marker genes (see methods). **d**. UMAP clusters of astrocyte nuclei from individual uninjured and SCI replicates. **e,f**. UMAP feature plots of selected DEGs. **g**. UMAP comparison of all uninjured and SCI astrocyte nuclei. **h**. Comparison of expression levels and percent of expressing nuclei of various marker genes enriched in and associated with different cell types in our final cohort of astrocyte nuclei. Because of astrocytes and ependyma share considerable gene expressions, we carefully excluded ependymal nuclei. Note that our uninjured and SCI astrocyte nuclei exhibited essentially undetectable expression for three highly and broadly expressed mouse spinal cord ependymal genes^88^, *Dnah12, Pifo, Odf3b*, and conversely our astrocyte nuclei broadly expressed high levels of multiple DEGs associated with uninjured or SCI astrocytes including *Gpc5, A2m, C3, Serpina3n*, and *Spp1* that are not at all, or only minimally, expressed by healthy or SCI-associated mouse spinal cord ependyma^85^. **i**. Comparison of changes in astrocyte transcription as detected by using RiboTag or snRNAseq at 28 days after SCI in different gene cohorts that were significantly detectable in both data sets: all 2092 snRNAseq significantly detected DEGs, 190 cAEGs, and 46 cARGs. Note the highly significant correlation of findings with both methods, including DEGs highlighted in the main text. **j-k**. UMAP clustering (**j**) and graph showing proportion of nuclei from uninjured or SCI mice per cluster (**k**) of astrocyte nuclei from lower thoracic spinal cord of uninjured mice and mice at two months after SCI, from another study, Skinnider et al.^30^, which show that in this data set, reactive astrocytes at two months after SCI also show clear separation from uninjured astrocytes in UMAP space. **l**. Comparison of changes in astrocyte transcription as detected by using snRNAseq at 28 days after SCI from the present study (O’Shea et al.) and at two months after SCI from Skinnider et al.^30^ in the 62 cAEGs and cARGs that were significantly detectable in both data sets. Note the highly significant correlation of findings by both methods, including DEGs highlighted in the main text. P-values for correlation coefficients (r) in (**i**) and (**l**) are calculated by two-sided test of the null hypothesis that r = 0.

We conducted snRNA-seq and immunohistochemical protein analyses to look for phenotypic features of mature lesion border astrocytes compared with astrocytes in healthy spinal cord. We isolated and sequenced 139,981 nuclei from the same region of thoracic spinal cord in uninjured mice (*n* = 4) and at 28 days after SCI (*n* = 4) when borders are mature (Fig. 7a). Nuclei were filtered and annotated using panels of multiple cell-type-specific marker genes, and clusters identified as astrocytes were extracted for further processing (Extended Data Fig. 8a–c). Re-clustering and further extraction based on multiple astrocyte marker genes confidently identified 15,637 astrocyte nuclei that were used for final analyses (Fig. 7a–e and Extended Data Fig. 8d–h). Expression changes among astrocyte genes detected by both snRNA-seq and RiboTag at 28 days after SCI were significantly correlated, including DEGs highlighted in our analyses (Extended Data Fig. 8i).

Uniform manifold approximation and projection (UMAP) analysis showed clear separation of uninjured and SCI-derived nuclei (Fig. 7b–e). A similar clear separation was also found in another study^30^ in an independent UMAP evaluation of astrocyte nuclei derived from a multicellular evaluation of lower thoracic spinal cord nuclei of uninjured mice and mice at 2 months after SCI (Extended Data Fig. 8k,l). Moreover, expression changes among consensus astrocyte genes detected by the present study at 28 days after SCI and by the previous study^30^ at 2 months after SCI were significantly correlated (Extended Data Fig. 8k,l).

**Extended Data Fig. 9:**
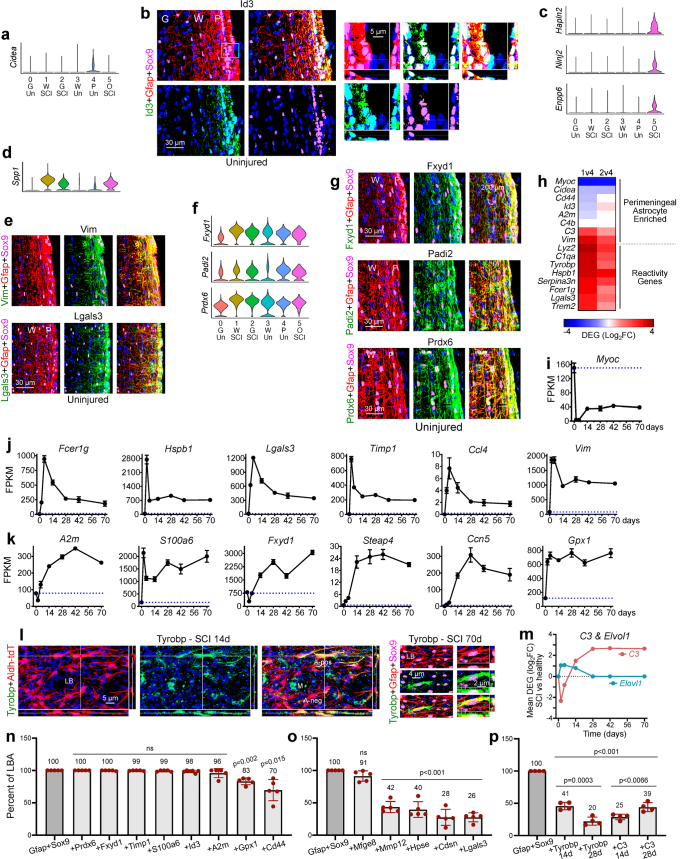
Molecular characteristics of perimeningeal astrocytes (PMA) and mature lesion border astrocytes (LBA). **a**. Violin plot of *Cidea* expression limited to cluster 4 of uninjured PMA (P). **b**. Immunohistochemistry shows Id3 protein levels are high in PMA (P), low in white matter astrocytes (W) and low or not detectable in grey matter astrocytes (G). **c**. Violin plots show OPC lineage markers *Hapln2, Ninj2, Enpp6* expression limited to cluster 5 of OPC-derived LBA (O). **d**. Violin plot shows high expression of *Spp1* (which encodes the cytokine and axon growth promoting molecule, osteopontin), in reactive astrocyte clusters 2 and 3 after SCI, and moderately enriched *Spp1* expression in uninjured perimeningeal astrocytes in cluster 4 compared with grey or white matter astrocytes in clusters 0 and 3, respectively. **e**. Immunohistochemistry shows Vim and Lgals3 protein levels are highly enriched in PMA (P). **f**. Violin plots show enriched expression of shows *Fxyd1*, *Padi2* and *Prdx6* in reactive astrocyte clusters (1,2,5) and PMA (5) compared with uninjured grey (0) or white (3) matter astrocytes. **g**. Immunohistochemistry shows Fxyd1, Padi2 and Prdx6 protein levels are enriched in PMA (P) relative to white matter astrocytes. **h**. Heatmap comparing expression levels in cluster 1 (reactive astrocytes) with cluster 4 (uninjured PMA) of DEGs enriched in PMA (top) or astrocyte reactivity DEGs (bottom). **i**. Astro-RiboTag RNAseq shows an acute and persisting decline in *Myoc* expression after SCI. **j,k**. Additional selected astrocyte DEGs exhibiting different patterns of expression changes in the form of acute rise followed by decline (i) or delayed but persistent increase (j) after SCI as detected by Astro-RiboTag RNAseq. **l**. Images of the astrocyte lesion border (LB) at 14 or 70 days after SCI immunohistochemically stained for Tyrobp and Aldh1l1-tdT-labelled astrocytes. The first set of orthogonal images at 14 days after SCI shows that about half of the tdT-positive astrocytes express clearly detectable levels of immunoreactive Tyrobp (A-pos) and that these are intermingled with other astrocytes do not express detectable Tyrobp (A-neg) or express only much lower levels, and tdT-negative cells likely to be Tyrobp-positive presumptive macrophages or microglia (M). The second set of survey and detail images show high Tyrobp expression in one tdT-positive astrocyte among other astrocytes that do not express detectable Tyrobp. **m**. Graph shows that the temporally dependent changes in the expression of *C3* and *Elvol1* occur in opposite directions after SCI. **n-p**. Cell counts of multiple immunohistochemically detected proteins co-localized in cell bodies or processes of Gfap+Sox9 expressing lesion border astrocytes (LBA) at different times after SCI. Values are expressed as the percentage of cells exhibiting co-localization with Gfap+Sox9. One sample t test with hypothetical value = 100% of LBA, p < 0.05 indicates marker expression is significantly less than 100%, ns, nonsignificant. **n**. Proportions of mature LBA that exhibit clearly detectable immunoreactive levels of the indicated proteins at 28 days or longer after SCI. One sample t test with hypothetical value = 100% of LBA, p < 0.05 indicates marker expression is significantly less than 100%, ns, nonsignificant. n = 5 per group. **o**. Proportions of LBA that exhibit clearly detectable immunoreactive levels of the indicated proteins at 14 days or longer after SCI, a time point of their highest corresponding gene expression levels. One sample t test with hypothetical value = 100% of LBA, p < 0.05 indicates marker expression is significantly less than 100%. n = 5 per group. **p**. Examples of two proteins that exhibit significant differences in the proportion of LBA that express detectable immunoreactivity at 14d or 28d after SCI. Two-way ANOVA with multiple comparison test by Tukey. n = 4 per group. Line plots in (i-k, m) are mean values +/- SEM where n = 4 mice for uninjured and all post SCI timepoints except 2d which was n = 5. Source data

In our present data, we categorized six major clusters of astrocytes: three from uninjured and three from SCI (Fig. 7b–e). Canonical markers of healthy and reactive astrocytes such as *Gfap* and *Aqp4* were expressed throughout all clusters (Fig. 7d). Consensus markers discriminated healthy gray matter (cluster 0) and white matter (cluster 3) astrocytes and identified separate major clusters of reactive astrocytes with transcriptional features associated with gray matter (cluster 2) and white matter (cluster 1) astrocytes (Fig. 7c–g and Extended Data Fig. 8e,f). Healthy spinal cord astrocytes also separated into a third cluster (cluster 4) with enriched expression of molecules such as *Myoc*, *Cidea* and *Id3* (Fig. 7c,h and Extended Data Fig. 9a) that have previously been associated with astrocytes that form so-called limitans borders adjacent to meninges^42,59,60^. IHC confirmed robust protein expression of Myoc and Id3 proteins in healthy perimeningeal astrocytes (Fig. 7m and Extended Data Fig. 9b). An additional small cluster (cluster 5) expressed reactive astrocyte markers and was enriched for OPC lineage markers *St18*, *Sox10*, *Plp1*, *Hapln2*, *Ninj2* and *Enpp6* (Fig. 7c,g,i and Extended Data Fig. 9c), suggesting that these cells may have derived from OPCs, although further experiments will be needed to directly test this possibility. Nevertheless, our findings provide both snRNA-seq and lineage tracing (Fig. 1 and Extended Data Fig. 1) evidence consistent with previous observations^34–37^ that local OPCs can give rise to a small contingent of lesion border astrocytes, as also noted in another recent single-cell RNA-seq study^61^.

We next evaluated how different gene cohorts consisting of all DEGs, cAEGs, cARGs or dARGs changed in the different major astrocyte clusters after SCI. PCA analyses showed that in all cases, gray matter astrocytes exhibited the greatest changes at 28 days after SCI relative to healthy, including markedly reduced expression of cAEGs, and markedly upregulated cARGs and dARGs (Fig. 7j). By contrast, white matter astrocytes exhibited minimal changes in gene expressions except for markedly upregulating cARGs (Fig. 7j).

**Fig. 8:**
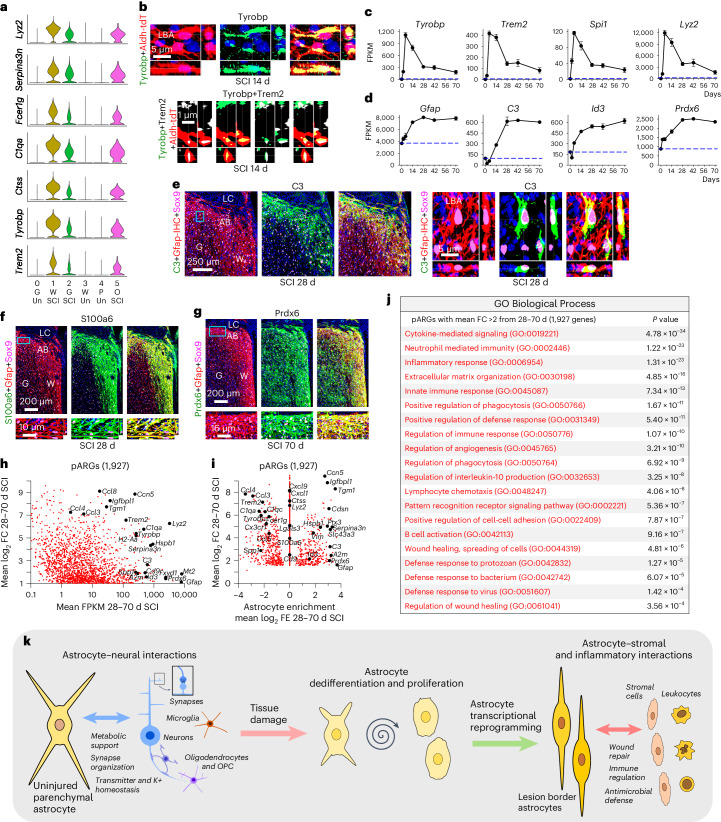
Features of mature LBAs. **a**, Violin plots of snRNA-seq detected genes enriched in reactive astrocyte clusters compared with uninjured. **b**, IHC of proteins Tyrobp and Trem2 in LBAs after SCI. **c**,**d**, Selected astrocyte DEGs exhibiting different patterns of expression changes in the form of acute rise followed by decline (**c**) or delayed but persistent increase (**d**) after SCI as detected by Astro-RiboTag RNA-seq. **e**,**f**,**g**, IHC of proteins C3 (**e**), S100a6 (**f**) and Prdx6 (**g**) in LBAs after SCI. Boxes show locations of expanded regions. **h**, Scatterplot comparing mean log_2_ FC and mean FPKM of 1,927 pARGs upregulated at least twofold from 28 to 70 days after SCI as detected by Astro-RiboTag RNA-seq. **i**, Scatterplot comparing mean log_2_ FC and mean log_2_ FE of 1,927 pARGs upregulated from 28 to 70 days after SCI. **j**, GO-BPs associated with 1,927 pARGs upregulated at least twofold from 28 to 70 days after SCI. **k**, Summary schematic showing local astrocyte responses to CNS tissue damage by dedifferentiation, proliferation and transcriptional reprogramming into border-forming wound repair astrocytes. Line plots are mean values; error bars, s.e.m.; *n* = 4 mice for uninjured and all post-SCI timepoints except day 2 (*n* = 5). *P* values in (**j**) calculated by two-sided Fisher’s exact test.

Historical evidence has suggested similarities between astrocyte borders around CNS lesions and limitans astrocytes adjacent to meninges^4,62–64^. We therefore looked for potential similarities and differences. Notable DEGs enriched in both healthy perimeningeal astrocytes (cluster 4) and lesion border reactive astrocytes (clusters 1, 2 and 5) compared with healthy gray or white matter astrocytes (clusters 0 and 3) included *Id3*, *Cd44, C3, C4b, A2m, Gpx1, Lxn, Vim, S100a6, Lgals3, Spp1, Fxyd1, Padi2* and *Prdx6* (Fig. 7g,h,k–p and Extended Data Fig. 9b,d–g). Both *Myoc* and *Cidea* were selective for healthy perimeningeal astrocytes and were essentially not detected in reactive astrocytes or in healthy gray matter or white matter astrocytes (Fig. 7h,m and Extended Data Fig. 9a). Conversely, healthy perimeningeal astrocytes did not express appreciable levels of multiple markers associated with overt astrocyte reactivity, including *Lyz2, Serpina3n, Fcerg1, Ctss, Trem2, Tyrobp, C1qa, Hsbp1* and others (Fig. 8a, Extended Data Figs. 8f,g and 9h). Reactive perimeningeal astrocytes were not discriminated as a separate cluster, most likely because they become indistinguishable from other reactive astrocytes owing to the pronounced and persistent downregulation of *Myoc* and other genes that distinguish them (Extended Data Fig. 9i). As indicated by our Astro-RiboTag data, certain reactivity DEGs peaked acutely from 2 to 14 days and then declined by 28 days but continued to be expressed thereafter at levels higher than in healthy astrocytes, such as *Tyrobp, Trem2, Spi1, Lyz2, Lgals3, Ccl4* and others (Fig. 8b,c and Extended Data Fig. 9j), and DEGs with this pattern were generally not shared with perimeningeal astrocytes (Fig. 8a and Extended Data Fig. 9h). By contrast, certain other reactivity DEGs only reached their peak expression at later times, generally after 14 days, and then persisted at those levels, and this pattern associated with mature lesion border astrocytes was particularly exhibited by DEGs such as *Gfap*, *C3*, *Id3*, *S100a6*, *Prdx6*, *Fxyd1*, *A2m* and others, which were also enriched in perimeningeal astrocytes that also form borders to stromal cells of the meninges (Figs. 7k–p and 8d–g and Extended Data Fig. 9f,g,k). These findings indicate that healthy perimeningeal astrocytes exhibit unique features that distinguish them from healthy gray matter and white matter astrocytes and share some features with reactive astrocytes but do not exhibit overt reactivity profiles.

*Trem2* and *Tyrobp* were not detectably expressed in healthy astrocytes but were robustly upregulated by reactive astrocytes after SCI as detected by both snRNA-seq and Astro-RiboTag RNA-seq, and we confirmed protein expression by IHC (Fig. 4c,f and 8a–c,h and Extended Data Figs. 4e, 8f and 9l). We have previously shown that reactive astrocytes can adopt gene expressions that are unexpected based on their absence in healthy CNS, such as the transcription factor *Spi1* (ref. ^17^) (Fig. 8c). *Trem2, Tyrobp* and *Spi1* are widely regarded as uniquely expressed by microglia in the CNS^65,66^ but can be expressed by reactive astrocytes, albeit at levels lower than other cells (Fig. 8i).

The antimicrobial defense factor *C3* (refs. ^49,50^) became persistently upregulated by over sixfold from 28 to 70 days in astrocytes after SCI, and immunoreactive protein was highly concentrated in lesion border astrocytes (Figs. 5e,f and 8d,e). C3 has been proposed as a marker of a subtype of reactive astrocytes that are neurotoxic to neurons across multiple CNS disorders, and generation of saturated lipids by astrocyte Elovl1 has been proposed as the mediator of their neurotoxicity^67^. We noted here that *C3* is expressed by certain astrocytes in healthy tissue, particularly perimeningeal and scattered white matter astrocytes, and that astrocyte *C3* expression declines acutely after SCI during periods when neurons are lost, after which *C3* becomes highly and persistently upregulated in many border-forming astrocytes that separate inflamed tissue lesions from immediately adjacent surviving neurons (Figs. 7o and 8d,e and Extended Data Fig. 8e). In addition, *C3* and *Elovl1* expression levels changed in opposite directions over time after SCI (Extended Data Fig. 9m). These findings suggest that *C3* may be expressed by astrocytes to exert natural antimicrobial functions^49,50^ around sites of tissue damage and that *C3* expression is not necessarily an absolute indicator of a neurotoxic reactive astrocyte phenotype.

Qualitative and quantitative IHC at single-cell resolution revealed considerable heterogeneity among lesion border astrocytes. Some immunoreactive proteins, such as Gfap, Prdx6, Fxyd1, Timp1, S100a6, Id3, A2m, Gpx1 and Cd44, were detectable at persistently high levels in all or most mature lesion border astrocytes from 28 to 70 days after SCI (Figs. 4d, 6n, 7p and 8f,g and Extended Data Figs. 4b, 7e and 9n). By contrast, other proteins, such as Mmp12, Hpse, Cdsn, Lgals3, H2-ab1, Cd74, C3 and Tyrobp, were detectable only in variable subsets of lesion border astrocytes that were intermingled with others with undetectable levels (Fig. 5h and 8e and Extended Data Figs. 4b, 5h, 6b and 9l,o,p). Moreover, detectable levels of immunoreactive protein for certain molecules showed temporal variation after SCI. For example, the number of border-forming astrocytes exhibiting detectable immunoreactive Tyrobp declined from 41% at 14 days after SCI to 20% or fewer at 28 days after SCI (Extended Data Fig. 9l,p), a trend that mimicked the decline in *Tyrobp* gene expression (Fig. 8c). Notably, those astrocytes expressing immunoreactive Tyrobp did so at a high level (Extended Data Fig. 9l). By contrast, the number of border-forming astrocytes exhibiting detectable immunoreactive C3 increased from 14 to 28–70 days (Extended Data Fig. 9p), similar to the increase in *C3* gene expression over time (Fig. 8c,d).

**Extended Data Fig. 10:**
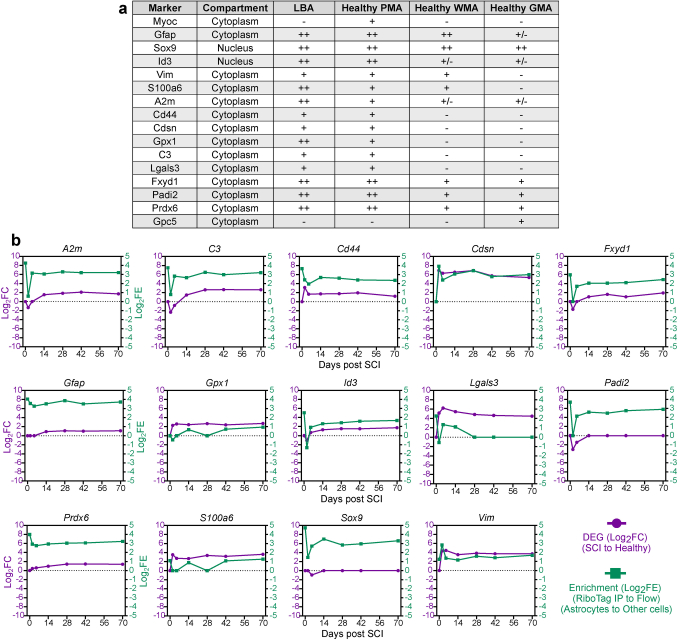
Comparisons of protein immunohistochemistry, DEG changes over time after SCI and DEG enrichment in astrocytes compared with other cells, for various astrocyte associated molecules, including potential markers enriched in mature border-forming wound repair astrocytes. **a**. Semi-quantitative (++,+,+/−, or −) observer scored comparison of immunoreactivity levels of various molecular markers across lesion border astrocytes (LBA) and astrocytes in healthy CNS tissue, perimeningeal astrocytes (PMA), grey matter astrocytes (GMA) and white matter astrocytes (WMA). **b**. Graphs compare changes in log_2_FC with changes in mean log_2_FE after SCI of DEGs encoding the molecular markers of LBA shown in a. Note that even though DEG expression of potential markers may be enriched in LBA compared with other cells, protein expression is in many cases not exclusive to LBA, and protein is often expressed also by adjacent non-neural cells in lesion cores, such as for C3, Lgals3, S100a6, Vim and others. Thus, these markers are not exclusive to LBA, and for this reason, co-detection of these markers together with Gfap and Sox9 as done here is essential to reliably identify LBA.

Certain DEGs, such as the calcium binding protein *S100a6*, that are not expressed by healthy gray matter astrocytes became highly upregulated by lesion border astrocytes that exhibit gray matter features (cluster 2), and immunoreactive S100a6 protein was prominent in astrocyte borders along lesions adjacent to spared gray matter (Figs. 7k and 8f and Extended Data Fig. 9k). Other DEGs, such as the antioxidant *Prdx6* and the intercalated disc protein *Fxyd1*, that were moderately expressed by healthy gray and white matter astrocytes as well as perimeningeal astrocytes, became prominently and persistently upregulated by lesion border astrocytes (Fig. 8d,g and Extended Data Fig. 9f,g,k). Notably, lesion border astrocytes and perimeningeal astrocytes shared prominent expression of various immunoreactive proteins, such as Cd44, A2m, Id3, C3, S100a6 and Gpx (Fig. 7k–p), that were expressed at low or undetectable levels in healthy gray matter or white matter astrocytes and therefore have the potential to serve as combinatorial markers of border astrocytes (Extended Data Fig. 10a,b).

To identify phenotypic features preferentially associated with mature reactive lesion border astrocytes compared with other cells in the lesion area, we examined a cohort of 1,927 DEGs persistently upregulated by more than twofold from 28 to 70 days after SCI in our Astro-RiboTag data, which we refer to as persisting astrocyte reactivity genes (pARGs) (Fig. 8h and Supplementary Data 3). We compared the enrichment of pARG transcripts in astrocytes versus other cells (Fig. 8i). The majority of the 1,927 pARGs that were highly and persistently upregulated by border astrocytes were either expressed at equal (1,330 pARGs) or greater (335 pARGs) levels by other cells, including *Tyrobp, Trem2, Lyz2, Lgals3, Cxcl1, Cxcl9, Ccl3, Ccl4, C1qa, C1qc, Fcer1g* and *Spp1* (Fig. 8c,i and Extended Data Fig. 9j). Nevertheless, 262 pARGs were both highly upregulated and highly enriched in astrocytes, including *Gfap, Tgm1, Ptx3, A2m, C3, Cdsn, Serpina3n, Hspb1, Id3, Timp1, Vim* and others (Fig. 8d,i and Extended Data Fig. 9k), suggesting that gene expression changes in astrocytes and other cells contribute to overlapping as well as differing functions. The top GO-BPs associated with all 1,927 pARG changes in astrocytes prominently included multiple functions associated with innate immune responses, regulation of inflammation, wound healing and antimicrobial defense (Fig. 8j).

## Discussion

In this Article, we show that after focal CNS tissue damage caused by SCI or stroke, local mature astrocytes dedifferentiate, proliferate and become transcriptionally reprogrammed into permanently altered new functional states. They downregulate molecules associated with homeostatic astrocyte–neuron interactions and upregulate molecules associated with wound healing, immune regulation and microbial defense (Fig. 8k). These wound repair astrocytes share morphologic and transcriptional features with perimeningeal limitans astrocytes and are the predominant source of neuroprotective borders that re-establish tissue integrity around CNS lesions by separating neural parenchyma from stromal and immune cells as occurs throughout the healthy CNS.

These findings have implications for understanding the diversity and complexity of astrocyte responses to injury and disease in different contexts. For example, increasing evidence indicates that astrocytes, for unknown reasons, attenuate homeostatic functions in aging and neurodegenerative disorders. Our findings indicate that the downregulation of astrocyte homeostatic functions is part of a natural wound repair response controlled by specific signaling mechanisms. Our findings suggest potential links of such responses to neurodegenerative conditions such as Alzheimer’s disease, in which astrocyte loss-of-functions and gain-of-functions may contribute to disease progression^68^ and in which the top 30 most consistently upregulated proteins across multiple studies included at least 11 molecules identified here not only as upregulated by, but also enriched in, wound repair astrocytes: Cd44, S100a6, Padi2, Prdx6, C3, Gpx1, Hsbp1, Gpnmb, Clu, Vim and Gfap^69^. Understanding the regulation of astrocyte reactivity responses in different contexts will help identify ways to selectively attenuate detrimental responses or augment beneficial ones. In this regard, we posit that the potential dysfunction of astrocyte wound repair responses is likely underestimated in CNS disorders. The identification here of additional protein markers associated with these cells may facilitate future neuropathological studies.

We demonstrate, by lineage tracing and transcriptional profiling, that over 90% of newly proliferated border-forming wound repair astrocytes derive from local Aldh1l1-expressing astrocytes, and we confirm, by lineage tracing and snRNA-seq, that about 10% derive from local OPCs^34–36^. Future studies using techniques such as RABID-seq^70^ or STICR^71^ will be useful to examine whether OPC-derived or astrocyte-derived border-forming cells contribute overlapping or differing functions. Ependymal cells have been proposed as the major source of lesion border astrocytes^72^, but this could not be confirmed by previous lineage-tracing studies, which showed that no meaningful number of border-forming astrocytes derived from ependymal cells after SCI^32^ or forebrain stroke^73^. Consistent with these observations, our lineage tracing here accounts for essentially all Gfap-positive and Sox9-positive lesion border astrocytes as derived from local astrocytes or OPCs.

The question arises as to why newly proliferated astrocytes form borders around CNS lesions as part of wound repair. Such astrocyte borders have long been proposed to comprise a ‘glial scar’ that contributes to inferior wound repair and regeneration failure. Preventing or removing these astrocytes was long regarded as a major goal to improve outcome. Challenging this notion, a large and accumulating body of evidence indicates that preventing or attenuating astrocyte border formation disrupts wound repair and causes increased spread of inflammation, failure of blood–brain barrier repair, increased loss of neural tissue, decreased functional recovery and in some cases increased mortality^4,17,18,23,24,26–29^, whereas augmenting astrocyte border formation reduces lesion size^23,30^.

In healthy CNS, all neural parenchyma is segregated from non-neural stromal cells either by astrocyte endfeet along blood vessels or by so-called limitans astrocytes that abut meningeal cells around the entire CNS. Structural similarities between perimeningeal astrocytes and lesion border astrocytes have been noted previously^4,62–64^. Here, we extend these observations by showing that lesion border astrocytes share molecular similarities with healthy perimeningeal astrocytes but also exhibit additional transcriptional profiles associated with wound repair and heightened levels of microbial defense and immune regulation. Previous histological observations have suggested that astrocytes surrounding CNS lesions form new limitans borders rather than scar tissue, particularly in human pathology^62–64^. Notably, true scar tissue derives from proliferating stromal cells that are unable to reconstitute lost parenchymal cell functions. In pro-regenerative mammalian organs, such as liver, intestinal epithelia and skin, injury induces parenchymal cell proliferation that enables tissue regeneration and recovery of organ function^74–76^. Proliferating parenchymal cells in these other organs are not considered to form scars; instead, scars are tissue formed by the proliferation of stromal cells when parenchymal regeneration is inadequate^74–76^. Astrocytes are key neural parenchymal cells that derive from the same neural stem cells as neurons^7^. Unlike mature neurons that are post-mitotic, mature astrocytes can re-enter the cell cycle and proliferate after injury. Newly generated and reprogrammed astrocytes around lesions exert a natural CNS parenchymal cell function when they surround and isolate stromal cell scar tissue and thereby re-establish CNS tissue integrity. In this manner, they preserve CNS function by separating functioning neural parenchyma from stromal and immune cells as occurs throughout the healthy CNS. These findings advocate that astrocyte borders around lesions should no longer be referred to as scars.

The appealing notion of potentially achieving scar-free wound healing in the adult CNS is supported by observations in mammalian neonates in which microglia and astrocytes sustain high levels of proliferation that enable a rapid glia-based repair rather than fibrotic repair, which is sufficient to promote neural circuit regeneration and recovery of function^77,78^. This may occur in part because neonatal astrocytes are already proliferative at the time of injury and may rapidly increase proliferation to replace lost neural parenchyma without stromal scarring in a manner similar to pro-regenerative organs. By contrast, astrocytes in mature CNS are dormant with respect to proliferation, and their proliferative response to injury is delayed, spatially restricted and transient. This response is adequate to generate new astrocyte borders around the rapidly formed stromal cell scars but is insufficient to rapidly achieve scar-free parenchymal repair. These observations support the pursuit of strategies that accelerate and extend astrocyte post-injury proliferative and immaturity states to augment neural parenchymal repair^23,30^ or rapidly replace lost glia^42^ in a variety of contexts associated with neural parenchymal loss.

## Methods

### Animals

Young adult male and female C57BL/6 mice between 2 and 4 months of age at the time of experimental procedure were used for all studies. For lineage tracing, Ai14 mice expressing the reporter tdT (JAX, 007914) were crossed with different Cre-driver lines: Aldh1l1-CreERT2 (ref. ^38^) (JAX, 031008), Pdgfra-CreERT^39^ (JAX, 018280) or NG2-CreERT^40,41^ (JAX, 008538). For sequencing of astrocyte ribosome-associated RNA, mice expressing RiboTag^33^ (JAX, 029977) were crossed either with Aldh1l1-CreERT2 (ref. ^38^) (JAX, 031008) or with mGfap-Cre-73.12 (ref. ^79^) (JAX, 012886). For postnatal astrocyte evaluations, mGfap-RiboTag mice were used at postnatal days P0, P3, P7, P14, P21, P35 and P63. Transgene expression for each sample was confirmed by genotyping of collected tail samples before processing for astrocyte-specific RNA. Mice were housed in a specific pathogen-free facility with a 12 h light–dark cycle and controlled temperature (20–25 °C) and humidity (50–70%) and were allowed free access to food and water. All in vivo experiments involving the use of mice were conducted according to protocols approved by the Animal Research Committee (ARC) of the Office for Protection of Research Subjects at the University of California Los Angeles (UCLA), under ARC numbers ARC-2017-044, ARC-2008-051, ARC-2015-073 and ARC-2000-001. Mice were assigned to experimental groups randomly although ensuring a balanced number of males and females. Animals were coded numerically, and all surgical procedures and subsequent analyses were conducted blind to experimental condition.

### Surgical procedures

All surgeries were performed under general anesthesia with isoflurane in oxygen-enriched air using an operating microscope (Zeiss) and rodent stereotaxic apparatus (David Kopf). All animals received the opiate analgesic buprenorphine (0.1 mg kg^−1^) subcutaneously before surgery and every 12 h for at least 48 h post surgery.

#### SCI

Laminectomy of a single vertebra was performed at cord level T10. A timed (5 s) lateral compression, complete crush SCI was made using No. 5 Dumont forceps (Fine Science Tools) with a tip width of 0.5 mm. Daily bladder expression was performed for the duration of experiments or until voluntary voiding returned.

#### Stroke

After a small craniotomy over the left coronal suture, 1.5 μl of L-NIO (N5-(1-iminoethyl)-l-ornithine) (cat. no. 0546, Tocris solution) (27.4 mg ml^−1^ in sterile PBS) was injected into the caudate putamen nucleus at 0.15 μl min^−1^ using target coordinates relative to bregma of +0.5 mm A/P, +2.5 mm L/M and −3.0 mm D/V by using a glass micropipette.

### Lineage tracing

Cell lineage tracing was conducted by using tdT reporter protein targeted to specific cells and temporally regulated via CreERT-loxP in transgenic mice^31^. tdT expression was activated in young adult mice by administering tamoxifen (Sigma, T5648-1G, 20 mg ml^−1^ in corn oil) by intraperitoneal injection (100 mg kg^−1^, once a day) for 5 days followed by clearance for 3 weeks before SCI or stroke, so that no residual tamoxifen remained. Using this approach, tdT becomes constitutively expressed by cells in which it has been activated by Cre during the period of tamoxifen delivery, and this expression is passed on to all progeny cells. Once tamoxifen is no longer administered and has cleared, only the originally targeted cells and their progeny express tdT.

### BrdU

BrdU (Sigma-Aldrich), 100 mg kg^−1^ day^−1^ dissolved in saline plus 0.007 N NaOH, was administered as single daily intraperitoneal injections on days 2 through 7 after SCI.

### Histology and immunohistochemistry

After terminal anesthesia by barbiturate overdose, mice were perfused transcardially with 4% paraformaldehyde (Electron Microscopy Sciences). Spinal cords were removed, post-fixed overnight and cryoprotected in buffered 30% sucrose for 48 h. Frozen sections of the spinal cord were prepared in the horizontal plane at 30 μm thickness using a cryostat microtome (Leica) and processed for immunofluorescence as previously described^27^.

#### Primary antibodies

Primary antibodies used included goat anti-A2m (1:300, AF1938; R&D Systems), rabbit anti-Aldh1l1 (1:1,000, Ab87117; Abcam), sheep anti-BrdU (1:800, NB-500-235; Novus), rat anti-C3 (1:400, NB200-540; Novus), goat anti-CD13 (1:600, AF2335; R&D Systems), rat anti-Cd44 (1:400, 14-0441-82; Invitrogen), rat anti-CD68 (1:1,000, MCA1957; Biorad), rabbit anti-Cd74 (1:200, A13958; Abclonal), rabbit anti-Cdsn (1:800,13184-1-AP; Proteintech), goat anti-Cxcl10 (1:200, AF-466; Novus), rabbit anti-Dnali1 (1:500, 17601-1-AP; Proteintech), rabbit anti-Fxyd1 (1:800, A15082; Abclonal), rabbit anti-GFAP (1:2,000, GA524, Z033401-2; Dako/Agilent), rat anti-GFAP (1:1,000, 13-0300; ThermoFisher), rabbit anti-hemagglutinin (1:1,000, H6908; Sigma-Aldrich), goat anti-Gpc5 (1:200, AF2607; R&D Systems), rabbit anti-Gpx1 (1:200, 29329-1-AP; Proteintech), goat anti-hemagglutinin (1:800, NB600-362; Novus Biologicals), rabbit anti-H2-Ab1 (1:200, A18658; Abclonal), rabbit anti-Hpse (1:200, 24529-1-AP; Proteintech), guinea pig anti-Iba1 (1:1,000, 234004; Synaptic Systems), rabbit anti-Iba-1 (1:800, 019-19741; Wako), rabbit anti-Id3 (1:500, 9837; Cell Signaling), rabbit anti-Kcnj10 (Kir4.1) (1:400, APC-035; Alomone Labs), rat anti-Lgals3 (1:200, 14-5301-82; ThermoFisher), rabbit anti-Lxn (1:500, 13056-1-AP; Proteintech), rabbit anti-Mfge8 (1:200, A12322; Abclonal), rabbit anti-Mmp12 (1:200, 22989-1-AP; Proteintech), goat anti-Myoc (1:400, AF2537; Novus), guinea pig anti-NeuN (1:1,000, 266004; Synaptic Systems), rabbit anti-NeuN (1:1,000, ab177487; Abcam), guinea pig anti-Olig2 (1:800, ABE1024; Millipore), rabbit anti-Olig2 (1:200, AB9610; Millipore), rabbit anti-Padi2 (1:300,12110-1-AP; Proteintech), rabbit anti-Prdx6 (1:500, 13585-1-AP; Proteintech), sheep anti-S100a6 (1:300, AF4584; R&D Systems), rabbit anti-S100a6 (1:200, A3461; Abclonal), goat anti-Serpina3n (1:200, AF4709; R&D Systems), goat anti-Sox9 (1:800, AF3075; R&D Systems), rabbit anti-Sox9 (1:800, 702016; ThermoFisher), goat anti-Sox10 (1:500, AF2864; R&D Systems), guinea pig anti-tdT (RFP) (1:1,500, 390-004; Synaptic Systems), rabbit anti-RFP (1:1,500, 600-401-379; Rockland), rabbit anti-Timp1 (1:800, 16644-1-AP; Proteintech), sheep anti-Trem2 (1:400, AF1729; Novus), rabbit anti-Tyrobp (1:400,12492S; Cell Signaling) and rat anti-Vim (1:200, MAB2105; Novus). All antibodies were sourced from commercial vendors and selected because they had previously been validated for fluorescent immunohistochemistry in mouse tissue and had a manufacturer-provided demonstration of specificity based on western blots and, in most cases, validation in peer-reviewed publications.

#### Fluorescence secondary antibodies

Alexa 488 (green), Cy3 (550, red) or Alexa 647 (far red) were used, all from Jackson Immunoresearch Laboratories. Mouse primary antibodies were visualized using the Mouse-on-Mouse detection kit (M.O.M., Vector). The nuclear stain was 4′,6′-diamidino-2-phenylindole dihydrochloride (DAPI, blue; 2 ng ml^−1^; Molecular Probes). Sections were cover-slipped using ProLong Gold anti-fade reagent (Invitrogen). Sections were examined and photographed with an epifluorescence microscope using structured illumination hardware and deconvolution software (Zeiss).

#### Quantification of positively stained cells

The number of lesion border astrocytes expressing specific individual immunoreactive proteins or incorporating BrdU was evaluated by using double and triple labeling immunohistochemistry combined with analysis of single cells in three dimensions. Stacks of optical slices were collected through the *z* axis (15–25 µm) in the astrocyte borders immediately adjacent to and within 250 µm of fibrotic lesion cores and were evaluated using the software Imaris 9.2 (Bitplane), Zen 3.1 (Zeiss) and ImageJ 1.53 (NIH). Values were expressed as the proportion of Gfap+Sox9-positive cells that were also positive for a given protein or BrdU. At least 30 cells were evaluated per animal examined.

### Fresh tissue collection and freezing for RiboTag or snRNA-seq

Uninjured mice and mice at various timepoints after complete crush SCI were perfused with ice-cold heparinized saline prepared using RNase-free and DNase-free water and 10× PBS for 2 min at 7 ml min^−1^ for blood clearance. Spinal cords were rapidly dissected on ice-chilled blocks. For SCI samples, the lesion core center was identified and a tissue block of 1 mm rostral and caudal was then rapidly removed. We have previously shown that astrocyte proliferation after SCI in mice is proportionally highest in the 250 µm zone immediately adjacent to the stromal cell lesion core and is almost as high in the next adjacent 250 µm zone and then tapers gradually over about 1 mm^13^. Our observations were equivalent here. Histological evaluations indicated that the lesion core extends rostro-caudally for about 500 µm and contains few astrocytes. Thus, the block of tissue collected here included the lesion core and about 750 µm of border tissue on either side of the lesion core, and the overwhelming majority of astrocytes in this tissue block will have been newly proliferated. We purposely made our dissections of tissue samples as small as possible to include primarily newly proliferated border-forming astrocytes while excluding nonproliferative astrocytes from adjacent spared neural tissue. Anatomically equivalent regions of spinal cord were taken from uninjured mice, including from postnatal samples. Tissue samples were rapidly snap-frozen in microcentrifuge tubes maintained in a dry ice bath and stored at −80 °C until further processing.

### RiboTag immunoprecipitation and RNA-seq

Frozen spinal cord tissue was processed by RiboTag immunoprecipitation^33^ using established methods^27,42^. In brief, tissue was homogenized in RiboTag lysis buffer and centrifuged to remove tissue debris. Immunoprecipitation of hemagglutinin-positive ribosomes was performed by incubating with anti-HA.11 Epitope Tag Antibody (Biolegend, 901515) for 4 h in a microcentrifuge tube on a microtube rotator kept at 4 °C. Immunoprecipitation solutions were combined with Pierce A/G Magnetic Beads (ThermoFisher, PI88803) and incubated overnight on a microtube rotator at 4 °C. On the second day, the solution was separated from the magnetic beads and processed as the ‘flow through’ sample representing mRNA of other cells not from RiboTag-positive cells. Magnetic beads were washed three times with high salt solution (50 mM Tris pH 7.4, 300 mM KCl, 12 mM MgCl_2_, 1% NP-40, 1 mM dithiothreitol (DTT), 100 mg ml^−1^ cyclohexamide). Unpurified RNA was collected from the magnetic beads by addition of RLT Plus buffer with BME and vigorous vortexing. RNA was then purified using RNeasy Plus Mini (for in vitro cell pellets) or Micro Kits (for spinal cord tissue) (QIAGEN, 74134 and 74034). Total mRNA derived from the RiboTag immunoprecipitation was quantified using a 2100 Bioanalyzer (Agilent), and RNA samples having RNA integrity numbers greater than seven were processed for RNA-seq. Sequencing was performed on poly-A selected libraries using Illumina NovaSeq S2 (UCLA Technology Center for Genomics & Bioinformatics) using paired-end reads (2 × 50 – 50 bp length) with an average of 50–100M reads per sample split over two lanes of the S2 flow cell.

### Transcriptomics analysis of RiboTag RNA-seq data

Analysis of RNA-seq raw data was performed in Galaxy using standardized workflows as previously described^42^. The R1 and R2 FASTQ files from lanes 1 and 2 that were obtained directly from the Illumina NovaSeq S2 run were concatenated and cleaned up using the Trimmomatic tool. Data were then aligned to the *M. musculus* (mm10) reference genome using the HISAT2 tool applying default parameters. Gene counts from the aligned datasets were performed using the featureCounts tool, applying default parameters. FPKM values were calculated for each gene directly in Excel (Microsoft) using standardized lists of gene lengths and normalization of the count data. DEG analysis on raw gene count data was conducted using Edge-R in Galaxy, applying Benjamini and Hochberg *P* value adjustment and TMM normalization. Across all studies, we used a conservative false discovery rate cutoff of <0.01 to define the significance of DEGs and evaluated at least four unique samples per experimental group. GO analyses were performed using the Enrichr tool (https://maayanlab.cloud/Enrichr). Differences in transcript expression across samples were evaluated using data-dimensionality reduction techniques, including principal component analysis and Euclidian distance as described below. Heatmaps of DEG data were generated using NG-CHM BUILDER (https://build.ngchm.net/NGCHM-web-builder). Violin plots of DEGs were generated using Prism 10.

### Threshold criteria for gene expression in healthy astrocytes

Healthy astrocyte expressed genes (AEGs) were defined as having an FPKM value greater than 0.1, which was a conservative cutoff that accounted for genes that were no more than one standard deviation below the mean of the log-transformed dataset and represented the upper 77% of all genes that had detectable counts in the dataset. Genes with an FPKM value at or above this threshold in healthy astrocytes were considered healthy expressed genes.

### Isolation and precipitation of astrocyte nuclei for snRNA-seq

Tissue samples were collected from *n* = 4 uninjured mice and *n* = 4 mice at 28 days after SCI and were rapidly frozen as described above. To avoid potential batch effects, frozen tissue samples were then all processed at the same time. To enrich for astrocyte nuclei, we used Sox9 antibody-binding and magnet-assisted nuclear immunoprecipitation using well-characterized procedures^80–82^ as follows. Tissue samples were first gently dissociated by trituration and pelleted by centrifugation. Nuclei were extracted from cell pellets by gentle resuspension in ice-cold lysis buffer (10 mM Tris buffer, 10 mM NaCl, 3 mM MgCl_2_, 0.1% Nonidet P40 Substitute). Nuclei were pelleted by centrifugation (model 5415R, Eppendorf; 500 r.p.m. for 5 min at 4 °C) and then resuspended in nuclei wash and resuspension buffer (1× PBS, 1% BSA, 0.2 U ul^−1^ RNase inhibitor) before being washed once more in nuclei wash and resuspension buffer and then filtered using a 5 ml polystyrene round-bottom tube with 35 µm cell-strainer cap and concentrated to a nuclei concentration of 1,000 nuclei per µl (1 × 10^6^ nuclei per ml), resuspended and incubated with Sox9 rabbit monoclonal antibody (ThermoFisher, 72016) for 30 min and then centrifuged at 700×*g* for 10 min. Pellets were resuspended in 80 μl of MACS buffer composed of 1× PBS (tissue culture grade; Ca^2+^, Mg^2+^ free), 0.5% nuclease-free BSA, and 2 mM EDTA, and then incubated with anti-Rabbit IgG Microbeads (Miltenyi, 130-048-602) for 20 min at 4 °C. After washing with 1 ml of MACS buffer at 300×*g* for 10 min at 4 °C, immunolabeled nuclei were enriched by magnetic separation using MACS MS columns (Miltenyi, 30-042-201, 130-042-102 and 130-042-303). Pellets were resuspended in an appropriate volume of resuspension buffer to achieve a final concentration of 1,000 nuclei per µl (1 × 10^6^ nuclei per ml) and immediately processed for library preparation using Chromium Next GEM Single Cell 3′ v3.1 kits following the manufacturer’s instructions. Eight library samples were processed simultaneously for RNA-seq on two lanes (lane 1: injured-1, injured-2, uninjured-1, uninjured-2; lane 2: injured-3, injured-4, uninjured-3, uninjured-4) of Illumina NovaSeq S4 with at least 300 M reads per library sample, conducted in-house by the UCLA Technology Center for Genomics & Bioinformatics using paired-end reads (2 × 100).

### Analysis of snRNA-seq data

Raw single-nuclei sequencing data was processed using RNA StarSolo on the Galaxy Single Cell Omics platform using *M. musculus* (mm10) reference genome, 3M-February-2018 barcode whitelist, the gencode vM25 annotation list and Cell Ranger v3 configure chemistry options. Scanpy tools were used through the Galaxy platform to convert genes, barcodes and matrix files derived from RNA StarSolo into an AnnData matrix h5ad format. Downstream analysis was performed in R (v4.1) using the Seurat package (v4). The dataset was pre-processed to remove cells with high mitochondrial reads, filter out genes that were detected in less than 200 cells and filter out low-quality cells that had less than 500 attributed genes. We then imposed stringent quality control metrics on unique molecular identifiers, the number of genes detected per cell and the proportion of mitochondrial reads (Extended Data Fig. 8b), thereby effectively mitigating the likelihood of doublet incorporation. Second, we used the CellBender algorithm^83^ to correct for ambient RNA contamination, a factor that can artificially inflate doublet rates. CellBender’s robust ambient RNA removal further contributed to the enhancement of data quality. Our prior investigations^84^ have revealed a lack of consensus among various doublet detection algorithms when identifying doublet cells in identical datasets. We therefore focused on rigorous upfront quality control measures and ambient RNA correction as more reliable avenues for enhancing data fidelity.

Next, we log_2_ normalized the dataset, identified the 4,000 most variable genes across the total population, computed principal components and summarized the top 50 principal components using the UMAP projection. Clustering of nuclei was performed on the UMAP data through Seurat using Louvain clustering algorithms with a resolution of 0.3, which resulted in 20 discrete clusters for nuclei from both uninjured and SCI tissue. Based on feature plot analysis of the distribution of marker genes in individual nuclei, clusters of nuclei were annotated as enriched for astrocytes, neurons, oligodendrocytes, OPCs or microglia on the basis of multiple cell-type-specific marker genes. Clusters broadly identified as enriched for astrocytes based on high expression of *Gfap, Aqp4, Slc1a2, Aldoc* and *Clu* with low or absent expression of markers for other cell types (Extended Data Fig. 8c) were extracted and re-clustered with a resolution of 0.3. We then selected clusters of astrocytes and eliminated clusters of other cell types based on panels of multiple consensus marker genes highly enriched in or selective for uninjured or reactive astrocytes^42^ or other specific cell types^85^. Examples of marker genes used to identify clusters included (but were not limited to) *Gfap*, *Aqp4*, *Gpc5, Slc7a10*, *Slc1a2*, *Slc4a4*, *Slc39a12*, *Fgfr3*, *Aldoc*, *Clu*, *Padi2*, *Fxyd1*, *Prdx6*, *Lcn2*, *Serpina3n*, *A2m*, *Spp1* and *C3* for uninjured or reactive astrocytes; *Dnah12*, *Pifo*, *Odf3b*, *Dynlrb2*, *Cfap126* and *Fam183b* for ependyma; *Pecam1*, *Ptprb*, *Adgrf5*, *Cytl1* and *Lmcd1* for endothelia; *Csf1r*, *P2ry12* and *Ptgs1* for microglia; *Ptprc* (*Cd45*) for leukocytes; *Mog* and *Cldn14* for oligodendroglia; *Pdgfra* and *Cspg4* (*Ng2*) for OPCs; *Pdgfra* for pericytes; *Fbn1* and *Lum* for stroma and fibroblasts; and *Syt1 and Stx1a* for neurons. Final selection of astrocyte clusters was based on high expression of multiple astrocyte markers combined with low or absent expression of markers of other cell types as shown for selected examples in Extended Data Fig. 8h. This process confidently identified 15,637 astrocyte nuclei from *n* = 4 uninjured mice (1,585, 1,576, 2,961 and 2,648 nuclei per mouse) and *n* = 4 SCI mice (1,967, 1,859, 1,709 and 1,332 nuclei per mouse) that were used for final analyses after re-clustering with a resolution of 0.15 (Extended Data Fig. 8d–g).

### PCA

PCA and Euclidean distance analysis were performed using XLStat (Addinsoft)^42^. For PCA presented throughout, the first two principal components were used to display two-dimensional scatterplots. PCA factor analysis to identify genes correlated with a particular PC used a factor loading threshold of >|0.8|. PCA data were represented as Euclidean distance plots throughout. Euclidean distance magnitude calculations were derived by assessing the vector magnitude in PCA space of a specific sample referenced to another sample as an initial point.

### Statistics, power calculations, group sizes and reproducibility

Graph generation and statistical evaluations of repeated measures were conducted by one-way or two-way ANOVA with Tukey’s post hoc independent pair-wise analysis or by one-sample *t*-tests in which the null hypothesis was equal to 100%, as appropriate, using Prism 10 (GraphPad Software). *P* values for GO evaluations were calculated by two-sided Fisher’s exact test using the Enrichr tool. Data distribution was assumed to be normal but this was not formally tested. Statistical details of experiments can be found in the figure legends including the statistical tests used and the number of replicative samples. Across all statistical tests, significance was defined as *P* < 0.05. Power calculations to determine group sizes were performed using G*Power Software v3.1.9.2. For immunohistochemical quantification analysis and RNA-seq, group sizes were calculated to provide at least 80% power when using the following parameters: probability of type I error (alpha) = 0.05, a conservative effect size of 0.25 and 2–5 treatment groups with multiple measurements obtained per replicate. All graphs show mean values ± s.e.m. as well as individual values as dot plots. All bar graphs are overlaid with dot plots in which each dot represents the value for one animal to show the distribution of data and the number (*n*) of animals per group. Lineage-tracing studies were repeated independently at least three times in different cohorts of mice across a 3-year period with similar results. All immunohistochemistry analyses were reproduced in at least *n* = 4 biological replicates.

### Reporting summary

Further information on research design is available in the Nature Portfolio Reporting Summary linked to this article.

## Online content

Any methods, additional references, Nature Portfolio reporting summaries, source data, extended data, supplementary information, acknowledgements, peer review information; details of author contributions and competing interests; and statements of data and code availability are available at 10.1038/s41593-024-01684-6.

### Supplementary information

Reporting Summary

Supplementary Data 1 Master lists of FPKMs of astrocytes and other cells (flow-through) used to calculate enrichment and DEGs (log_2_ (fold changes from healthy)).

Supplementary Data 2 Lists of DEGs in named gene cohorts: Fig. 2b, all DEGs (13,527); Fig. 2d, healthy astrocyte EGs (15,722); Fig. 2d, newly expressed genes (889); Fig. 3b, consensus healthy astrocyte expressed genes (cAEGs, 429); Fig. 3g, immune-precipitate healthy astrocyte expressed genes (ipAEGs, 2,806); Fig. 3i,j, unsupervised proliferation genes (91); Fig. 4a–c, consensus reactivity genes (cARGs, 170); Fig. 4f, DEGs more than twofold upregulated at all times (1,129).

Supplementary Data 3 Lists of DEGs in named gene cohorts: Fig. 5a, inflammation DEGs (1,708); Fig. 6b, EMT genes (197); Fig. 6e, wound healing genes (278); Fig. 6h, cell adhesion genes (425); Fig. 6k, delayed astrocyte reactivity DEGs (dARGs, 757); Fig. 8h–j, persisting astrocyte reactivity DEGs (pARGs, 1,927).

### Source data

Source Data Fig. 1 Statistical Source Data

Source Data Fig. 2 Statistical Source Data

Source Data Extended Data Fig. 1 Statistical Source Data

Source Data Extended Data Fig. 2 Statistical Source Data

Source Data Extended Data Fig. 9 Statistical Source Data

## Data Availability

Raw FASTQ sequencing files and processed count data have been deposited at Gene Expression Omnibus and are publicly available with accession number GSE241628 for RiboTag data, accession number GSE247844 for our snRNA-seq data from this study and GSE234774 for snRNA-seq data from a previous publication^30^. All data generated for this study are included in the main and extended data figures and supplementary data files with lists of different gene cohorts examined. For all quantitative figures, source data are provided in Supplementary Information. Other data that support the findings of this study are available on reasonable request from the corresponding authors. Source data are provided with this paper.
