# Supplementary material for: Derivation and transcriptional reprogramming of border-forming wound repair astrocytes after spinal cord injury or stroke in mice
Source: Nat Neurosci. 2024 Jun 21;27(8):1505–21. doi: 10.1038/s41593-024-01684-6 (PMC11303254; doi:10.1038/s41593-024-01684-6)
Supplement: Supplementary file 1 — Reporting Summary [file 41593_2024_1684_MOESM1_ESM.pdf]

nature portfolio

Corresponding author(s): Michael Sofroniew & Timothy O'Shea

Last updated by author(s): 10 May 2024

Reporting Summary

Nature Portfolio wishes to improve the reproducibility of the work that we publish. This form provides structure for consistency and transparency in reporting. For further information on Nature Portfolio policies, see our Editorial Policies and the Editorial Policy Checklist.

Statistics

For all statistical analyses, confirm that the following items are present in the figure legend, table legend, main text, or Methods section.

n/a	Confirmed	
☐	☒	The exact sample size (*n*) for each experimental group/condition, given as a discrete number and unit of measurement
☐	☒	A statement on whether measurements were taken from distinct samples or whether the same sample was measured repeatedly
☐	☒	The statistical test(s) used AND whether they are one- or two-sided<br>*Only common tests should be described solely by name; describe more complex techniques in the Methods section.*
☒	☐	A description of all covariates tested
☐	☒	A description of any assumptions or corrections, such as tests of normality and adjustment for multiple comparisons
☐	☒	A full description of the statistical parameters including central tendency (e.g. means) or other basic estimates (e.g. regression coefficient) AND variation (e.g. standard deviation) or associated estimates of uncertainty (e.g. confidence intervals)
☐	☒	For null hypothesis testing, the test statistic (e.g. *F*, *t*, *r*) with confidence intervals, effect sizes, degrees of freedom and *P* value noted<br>*Give P values as exact values whenever suitable.*
☒	☐	For Bayesian analysis, information on the choice of priors and Markov chain Monte Carlo settings
☒	☐	For hierarchical and complex designs, identification of the appropriate level for tests and full reporting of outcomes
☒	☐	Estimates of effect sizes (e.g. Cohen's *d*, Pearson's *r*), indicating how they were calculated

Our web collection on statistics for biologists contains articles on many of the points above.

Software and code

Policy information about availability of computer code

Data collection	Zen 3.1 (Blue Edition) (Zeiss).
Data analysis	NIH Image J (1.51), G*Power Software V 3.1.9.2., Microsoft Excel for Microsoft Office 365 ProPlus, Imaris 9.2 (Bitplane), or Zen 3.1 (Blue Edition) (Zeiss), Prism 10 (GraphPad Software Inc); XLStat Basic 2020.3.1 (Addinsoft Inc), Galaxy (https://usegalaxy.org/) to use the following tools - Trimmomatic (Galaxy Version.38.0), HISAT2 (Galaxy Version 2.1.0+galaxy4), featureCounts (Galaxy Version 1.6.3+galaxy2), EdgeR (Galaxy Version 3.34.0+galaxy1), ScanPy Galaxy Suite 1.1; Enrichr (https://maayanlab.cloud/Enrichr/).

For manuscripts utilizing custom algorithms or software that are central to the research but not yet described in published literature, software must be made available to editors and reviewers. We strongly encourage code deposition in a community repository (e.g. GitHub). See the Nature Portfolio guidelines for submitting code & software for further information.

Data

Policy information about availability of data

All manuscripts must include a data availability statement. This statement should provide the following information, where applicable:

- Accession codes, unique identifiers, or web links for publicly available datasets
- A description of any restrictions on data availability
- For clinical datasets or third party data, please ensure that the statement adheres to our policy

Raw FASTQ sequencing files and processed count data have been deposited at Gene Expression Omnibus (GEO) and are publicly available with Accession Number

GSE241628 for RiboTag data and Accession Number GSE247844 for our snRNAseq data, and GSE234774 for Skinnider et al data. All data generated for this study are included in the main and supplementary figures and Supplementary Information fiiles. For all quantitative figures, files of statistics source data of individual values as well as the results of statistical tests are provided with the paper. Other data that support the findings of this study are available on reasonable request from the corresponding authors.

Research involving human participants, their data, or biological material

Policy information about studies with human participants or human data. See also policy information about sex, gender (identity/presentation), and sexual orientation and race, ethnicity and racism.

Reporting on sex and gender	n/a
Reporting on race, ethnicity, or other socially relevant groupings	n/a
Population characteristics	n/a
Recruitment	n/a
Ethics oversight	n/a

Note that full information on the approval of the study protocol must also be provided in the manuscript.

Field-specific reporting

Please select the one below that is the best fit for your research. If you are not sure, read the appropriate sections before making your selection.

☒ Life sciences ☐ Behavioural & social sciences ☐ Ecological, evolutionary & environmental sciences

For a reference copy of the document with all sections, see nature.com/documents/nr-reporting-summary-flat.pdf

Life sciences study design

All studies must disclose on these points even when the disclosure is negative.

Sample size	For in vivo experiments, the animal group sizes were calculated to provide at least 80% power when using the following parameters: probability of type I error (alpha) = .05, a conservative effect size of 0.25, 2-5 treatment groups with multiple measurements obtained per replicate. For all experiments performed as part of this paper the groups sizes are reported.
Data exclusions	No data were excluded from analysis.
Replication	In vivo experiments that involved injection of stroke inducing agent L-NIO or application of SCI crush injury were repeated independently at least three times in different cohorts of mice across a three-year period with similar results. In addition, different cohorts of mice were evaluated with different techniques (RNAseq, ATACseq or protein detection) that yielded comparable and consistent results. Key data generated from the immunohistochemistry analysis were repeated independently by two co-authors. All replications were successful.
Randomization	Animals were randomly assigned numbers and thereafter were evaluated blind to experimental condition. Across all experiments animals were randomized for weight, age and sex. For in vitro studies, imaging fields of view were chosen randomly.
Blinding	Animals were randomly assigned numbers and thereafter were evaluated blind to experimental condition throughout RiboTag immunoprecipitation and RNA extraction, immunohistochemical processing and imaging.

Reporting for specific materials, systems and methods

We require information from authors about some types of materials, experimental systems and methods used in many studies. Here, indicate whether each material, system or method listed is relevant to your study. If you are not sure if a list item applies to your research, read the appropriate section before selecting a response.

Materials & experimental systems

n/a	Involved in the study
☐	☒ Antibodies
☒	☐ Eukaryotic cell lines
☒	☐ Palaeontology and archaeology
☐	☒ Animals and other organisms
☒	☐ Clinical data
☒	☐ Dual use research of concern
☒	☐ Plants

Methods

n/a	Involved in the study
☒	☐ ChIP-seq
☒	☐ Flow cytometry
☒	☐ MRI-based neuroimaging

Antibodies

Antibodies used

Goat anti-A2m (1:300, AF1938; R&D Systems); rabbit anti-Aldh1l1 (1:1000; Ab87117; Abcam, Cambridge, MA, USA); sheep anti-BrdU (1:800, NB-500-235; Novus); rat anti-C3 (1:400, NB200-540; Novus); goat anti-CD13 (1:600, AF2335; R&D Systems, USA); rat anti-Cd44 (1:400, 14-0441-82; Invitrogen); rat anti-CD68 (1:1000, MCA1957; Biorad, USA); rabbit anti-Cd74 (1:200, A13958; Abclonal); rabbit anti-Cdsn (1:800,13184-1-AP; Proteintech); goat anti-Cxcl10 (1:200, AF-466; Novus); rabbit anti-Dnali1 (1:500, 17601-1-AP, Proteintech); rabbit anti-Fxyd1 (1:800, A15082, Abclonal); rabbit anti-GFAP (1:2,000; GA524; Z033401-2; Dako/Agilent Tech., CA); rat anti-GFAP (1:1,000, 13-0300; Thermofisher, USA); rabbit anti-hemagglutinin (HA) (1:1000, H6908, Sigma); mouse anti-hemagglutinin (HA.11) (1:1000, 901515, Biolegend); goat anti-Gpc5 (1:200, AF2607; R&D Systems); rabbit anti-Gpx1 (1:200, 29329-1-AP; Proteintech); goat anti-HA (1:800, NB600-362, Novus Biologicals); rabbit anti-H2-Ab1 (1:200, A18658; Abclonal); rabbit anti-Hpse (1:200,24529-1-AP; Proteintech);guinea pig anti-Iba1 (1:1000, 234004; Synaptic Systems, USA); rabbit anti-Iba-1 (1:800, 019-19741; Wako, Osaka, Japan); rabbit anti-Id3 (1:500; 9837; Cell Signaling); rabbit anti-Kcnj10 (Kir4.1) (1:400, APC-035; Alomone labs)rat anti-Lgals3 (1:200, 14-5301-82; ThermoFisher); rabbit anti-Lxn (1:500, 13056-1-AP; Proteintech); rabbit anti-Mfge8 (1:200, A12322; Abclonal); rabbit anti-Mmp12 (1:200, 22989-1-AP; Proteintech); goat anti-Myoc (1:400, AF2537; Novus); guinea pig anti-NeuN (1:1000, 266004; Synaptic Systems); rabbit anti NeuN (1:1000, ab177487, Abcam); guinea pig anti-Olig2 (1:800, ABE1024; Millipore); rabbit anti-Olig2(1:200, AB9610; Millipore); rabbit anti-Padi2 (1:300,12110-1-AP; Proteintech); rabbit anti-Prdx6 (1:500, 13585-1-AP; Proteintech); sheep anti-S100a6 (1:300, AF4584; R&D Systems); rabbit anti-S100a6 (1:200, A3461; Abclonal); goat anti-Serpina3n (1:200, AF4709; R&D Systems); goat anti-Sox9 (1:800, AF3075; R&D Systems); rabbit anti-Sox9 (1:800, 702016; ThermoFisher); goat anti-Sox10 (1:500, AF2864; R&D Systems); guinea pig anti-tdT (RFP) (1:1500,390-004; Synaptic systems); rabbit anti-RFP (1:1500,600-401-379; Rockland); rabbit anti-Timp1 (1:800, 16644-1-AP; Proteintech); sheep anti-Trem2 (1:400, AF1729; Novus); rabbit anti-Tyrobp (1:400,12492S; Cell Signaling); rat anti-Vim (1:200, MAB2105; Novus).

Validation

All antibodies used were sourced from commercial vendors and were selected because they had previously been validated for use on mouse tissue (validated mouse reactivity) and for use in fluorescent immunohistochemistry (IHC) applications. Furthermore, validation of these antibodies can be found in peer reviewed publications by our team and others which are referenced throughout the manuscript. Additional validation information of each antibody is available from the various manufacturers' websites and validation information for relevant antibodies is provided below.
Rabbit anti-GFAP (1:1000; Cat#Z-0334, Dako, Santa Clara, CA) validated and used consistently over many publications (e.g. (Anderson et al. Nature. 2016 & 2018), validated by Dako and informationhe, used extensively across many other publications (~1839 citations on CiteAb);
Rat anti-GFAP (1:1000, Cat#13-0300, Thermofisher, Grand Island, NY) validated by company on their website and used within the concentration range recommended for IHC, cross-validated by us by comparing with the Rabbit GFAP above;
Rabbit anti NeuN (1:1000, Cat#Ab177487, Abcam, Cambridge, MA) - information describing validation of concentration and for use with mouse on company website, used at recommended concentration for IHC by manufacturer and used by us extensively in previous studies (O’Shea et al. Nature Communications. 2020);
Guinea pig anti-NeuN (1:1000, Cat#266-004, Synaptic Systems, Goettingen, Germany) validation information on company website, and our own successful validation was made by comparing with Rabbit anti-NeuN above.
Goat anti-CD13 (1:200, Cat#AF2335, R&D systems, Minneapolis, MN) - validation information on company website and used by us at recommended concentration range in previous studies (e.g. O’Shea et al. Nature Communications. 2020);
rabbit anti-Fibronectin (1:500, Cat#AB2033, Millipore, Burlington, MA) - validation information on company website and used by us extensively in previous studies (e.g. Anderson et al. Nature. 2018);
Rat anti-Galectin-3 (1:200, Cat#14-5301-82 Invitrogen-Thermofisher Scientific, Grand Island, NY)- validation information on company website, manufacturer states that "Antibody was verified by Knockdown to ensure that the antibody binds to the antigen stated" and used at the specified concentration for IHC provided by the manufacturers;
Rat anti-CD68 (1:1000, Cat# MCA1957, AbDserotec-BioRad, Hercules, CA)-validation information on company website and used previously by us in publications;
Rabbit anti-Iba-1 (1:800, Cat#019-19741, Wako, Osaka, Japan)-validation information on company website and used previously by us in publications;
Guinea pig anti-Iba-1 (1:800, Cat#234-004, Synaptic systems, Goettingen, Germany)-validation information on company website, staining profile was compared with the Rabbit anti-Iba-1 to confirm specificity;
Rabbit anti-P2Y12R (1:500, Cat#AS-55043A, Anaspec, Fremont, CA)-validation information on company website, used within recommended concentration, staining compared with Iba-1 in uninjured mouse brain tissue to confirm specificity for microglia;
Rabbit Hemagglutinin (HA) (1:1000, Sigma #H6908) and mouse anti-hemagglutinin (HA.11) (1:1000, 901515, Biolegend); validation information on company websites, used within recommended concentrations, staining and immun-precipitation compared with previous studies (e.g. Anderson et al. Nature. 2016);
Goat HA (1:800, Novus, NB600-362) validation information on company website, used within recommended concentration, staining compared with Rabbit HA above;
goat anti-Nestin (1:500, R&D, AF2736) validation information on company website, used within recommended concentration, used previously by us (Wollenberg et al. 2018. Biomaterials).
goat anti-Sox9 (1:500, R&D systems, AF3075) information describing validation of concentration and for use with mouse on company

website, used at recommended concentration for IHC by manufacturer and used by us extensively in previous studies (Ren et al. Scientific Reports. 2017);
rabbit Aldh1l1 (1:1000, Abcam, Ab87117) information describing validation of concentration and for use with mouse on company website, used at recommended concentration for IHC by manufacturer and used by us extensively in previous studies (Ren et al. Scientific Reports. 2017);
goat anti-Clusterin (Clu) (1:200, R&D systems, AF2747) information describing validation of concentration and for use with mouse on company website, used at recommended concentration for IHC by manufacturer;
Rabbit anti-Tuj-1 (1:500, Sigma, T2200-200UL) validation information on company website, used within recommended concentration, used previously by us (Wollenberg et al. 2018. Biomaterials).
rat anti-Vimentin (1:200; R&D Systems, MAB2105) information describing validation of concentration and for use with mouse on company website, used at recommended concentration for IHC by manufacturer;
rat anti-Cd44 (IM7) (1:200; Thermofisher Scientific, #14-0441-82) information describing validation of concentration and for use with mouse on company website, used at recommended concentration for IHC by manufacturer;
Rabbit anti-Id3 (1:200; Cell Signaling Technology, #9837) information describing validation of concentration and for use with mouse on company website, used at recommended concentration for IHC by manufacturer.

Animals and other research organisms

Policy information about studies involving animals; ARRIVE guidelines recommended for reporting animal research, and Sex and Gender in Research

Laboratory animals	All in vivo animal experiments were conducted within approved UCLA facilities using wildtype or transgenic C57/BL6 female and male mice that were aged between 8 weeks and four months old at the time of craniotomy or spinal cord injury surgery. For lineage tracing, Ai14 mice expressing the reporter, tdTomato (tdT) (JAX: 007914) were crossed with different Cre-driver lines: (1) Aldh1l1-CreERT2 (JAX: 031008), (2) Pdgfra-CreERT-tdT (JAX: 018280) or (3) NG2-CreERT-tdT(JAX: 008538). B6N.129-Rpl22tm1.1Psam/J (RRID: IMSR_JAX: 011029) 9RiboTag mice) were crossed either with Aldh1l1-CreERT2 (JAX: 031008) or with mGfap-Cre-73.12 (JAX: 012886). For postnatal astrocyte evaluations, mGfap-RiboTag mice were used at postnatal days P0, P3, P7, P14, P21, P35, P63. Transgene expression for each sample was confirmed by genotyping of collected tail samples prior to processing for astrocyte specific RNA. Mice were housed in a 12-hour light/dark cycle in a specific pathogen-free facility with controlled temperature (20°C-25°C) and humidity (50-70%) and were provided with food and water ad libitum.
Wild animals	No wild animals were used in the study.
Reporting on sex	Sex was considered in study design and animals of both sexes were examined (n=8 males and n = 8 females). Minimal differences were found as described in the text and data disaggregated for sex are shown in Extended Data Fig. 2l-n.
Field-collected samples	No field collected samples were used in the study.
Ethics oversight	All in vivo experiments involving the use of mice were conducted according to protocols approved by the Animal Research Committee (ARC) of the Office for Protection of Research Subjects at University of California Los Angeles (UCLA). ARC Numbers: ARC-2017-044; ARC-2008-051; ARC # 2015-073; ARC-2000-001.

Note that full information on the approval of the study protocol must also be provided in the manuscript.
